# Protease-dependent defects in N-cadherin processing drive PMM2-CDG pathogenesis

**DOI:** 10.1172/jci.insight.153474

**Published:** 2021-12-22

**Authors:** Elsenoor J. Klaver, Lynn Dukes-Rimsky, Brijesh Kumar, Zhi-Jie Xia, Tammie Dang, Mark A. Lehrman, Peggi Angel, Richard R. Drake, Hudson H. Freeze, Richard Steet, Heather Flanagan-Steet

**Affiliations:** 1 Complex Carbohydrate Research Center, University of Georgia, Athens, Georgia.; 2 JC Self Research Institute, Greenwood Genetic Center, Greenwood, South Carolina, USA.; 3 Sanford Children’s Health Research Center, Sanford Burnham Prebys Medical Discovery Institute, La Jolla, California, USA.; 4 Department of Pharmacology, UT Southwestern Medical Center, Dallas, Texas, USA.; 5 Department of Cell and Molecular Pharmacology and Experimental Therapeutics, Medical University of South Carolina, Charleston, South Carolina, USA.

**Keywords:** Development, Cartilage, Genetic diseases, Glycobiology

## Abstract

The genetic bases for the congenital disorders of glycosylation (CDG) continue to expand, but how glycosylation defects cause patient phenotypes remains largely unknown. Here, we combined developmental phenotyping and biochemical studies in a potentially new zebrafish model (*pmm2^sa10150^*) of PMM2-CDG to uncover a protease-mediated pathogenic mechanism relevant to craniofacial and motility phenotypes in mutant embryos. Mutant embryos had reduced phosphomannomutase activity and modest decreases in N-glycan occupancy as detected by matrix-assisted laser desorption ionization mass spectrometry imaging. Cellular analyses of cartilage defects in *pmm2^sa10150^* embryos revealed a block in chondrogenesis that was associated with defective proteolytic processing, but seemingly normal N-glycosylation, of the cell adhesion molecule N-cadherin. The activities of the proconvertases and matrix metalloproteinases responsible for N-cadherin maturation were significantly altered in *pmm2^sa10150^* mutant embryos. Importantly, pharmacologic and genetic manipulation of proconvertase activity restored matrix metalloproteinase activity, N-cadherin processing, and cartilage pathology in *pmm2^sa10150^* embryos. Collectively, these studies demonstrate in CDG that targeted alterations in protease activity create a pathogenic cascade that affects the maturation of cell adhesion proteins critical for tissue development.

## Graphical abstract

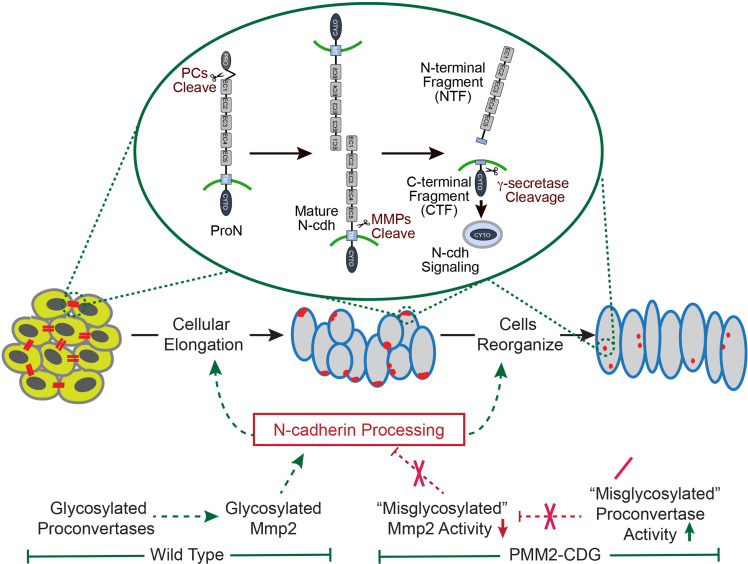

## Introduction

Congenital disorders of glycosylation (CDG) are a heterogeneous group of genetic diseases caused by defects in enzymes, transporters, and trafficking factors needed for protein and lipid glycosylation (1). The most common of the CDG, PMM2-CDG, results from variants in phosphomannomutase 2 (*PMM2*), encoding an enzyme that converts mannose-6-phospate (M6P) to mannose-1-phosphate (M1P) (2, 3). M1P is a precursor for guanosine diphosphate–mannose (GDP-mannose), a nucleotide sugar essential for the synthesis of lipid-linked oligonucleotide precursors needed for N-linked glycosylation (4). Defects in *PMM2* limit the production of GDP-mannose, causing reduced glycosylation of serum glycoproteins and numerous clinical manifestations. Common features include failure to thrive, neurological and cognitive impairment, and skeletal dysplasia (5). Despite nearly 4 decades of research on PMM2-CDG, the connection between hypoglycosylation of proteins and phenotypes remains enigmatic. To date no underglycosylated glycoprotein has been mechanistically linked to disease in an affected tissue. This barrier has created a major gap in our understanding of the molecular and cellular mechanisms driving CDG pathogenesis, and thus, has limited the development of therapies.

A major hurdle in defining CDG pathogenesis is the ability to identify sensitive glycoproteins beyond the classic markers, such as transferrin found in serum. Elucidating the pathogenic mechanisms associated with PMM2-CDG is further challenged by the difficulty in generating animal models that faithfully mimic the human disease. Complete loss of many N-glycosylation genes, particularly those involved in lipid-linked oligosaccharide biosynthesis, is lethal. Thus, complete gene knockout is not tenable. Early attempts to either knock out *PMM2* or knock in the most common human PMM2-CDG allele, p.Arg141His (p.Arg137His in mice), resulted in early embryonic lethality (6). In contrast, knockin of another common allele, p.Phe119Leu (p.Phe118Leu in mice), only mildly reduced enzymatic activity, yielding no detectable phenotypes (7). Compound heterozygous expression of both the p.Arg137His and p.Phe118Leu alleles was also embryonic lethal (7). More recently, another mouse harboring the most common genotype found in human patients, p.Arg141His;p.Phe119Leu (in this mouse model p.Arg137His;p.Phe115Leu), did survive postnatally (8). To circumvent the limitations of the mouse models, prior studies in zebrafish utilized morpholinos to transiently inhibit *pmm2* expression in developing embryos (9). Morphants deficient in *pmm2* exhibited altered craniofacial cartilage development and impaired motility that were consistent with human PMM2-CDG patients. These studies also showed increased M6P can diminish N-glycosylation precursors by enhancing the release of free glycan from lipid-linked oligonucleotide (LLO). Similar studies involving RNA interference–mediated (RNAi-mediated) inhibition of *pmm2* expression in Drosophila also demonstrated alterations in movement that were associated with abnormally formed neuromuscular synapses (10).

To date, no conclusive pathogenic mechanism has emerged that explains the clinical features in patients with PMM2-CDG. Using a powerful combination of developmental phenotyping and molecular investigation in a stable genetic PMM2-CDG zebrafish model (*pmm2^sa10150^*), we uncovered a protease-mediated defect in processing of the cell adhesion molecule N-cadherin. Altered N-cadherin processing is relevant to both the craniofacial and motility phenotypes affecting mutant embryos. Biochemical and molecular analyses demonstrated alterations in the activities of both proconvertases (PCs) and matrix metalloproteinases (Mmps) in *pmm2^sa10150^* mutant embryos. Using genetic and pharmacological approaches to manipulate PC activity, we showed that reducing PC activity restored normal Mmp activity, improving N-cadherin processing and cartilage pathology in *pmm2* embryos homozygous for the sa10150 allele (*pmm2^m/m^* embryos). These data provide a mechanistic link among protein PCs and Mmp-dependent N-cadherin processing and disease phenotypes. Together, these studies reinforce the concept that highly selective alterations in N-glycosylation in the context of one of the CDG create a pathogenic cascade that affects cell adhesion proteins critical for tissue development.

## Results

### Mutation of an essential splice site in the zebrafish pmm2 gene creates a hypomorphic allele.

**Figure 1:**
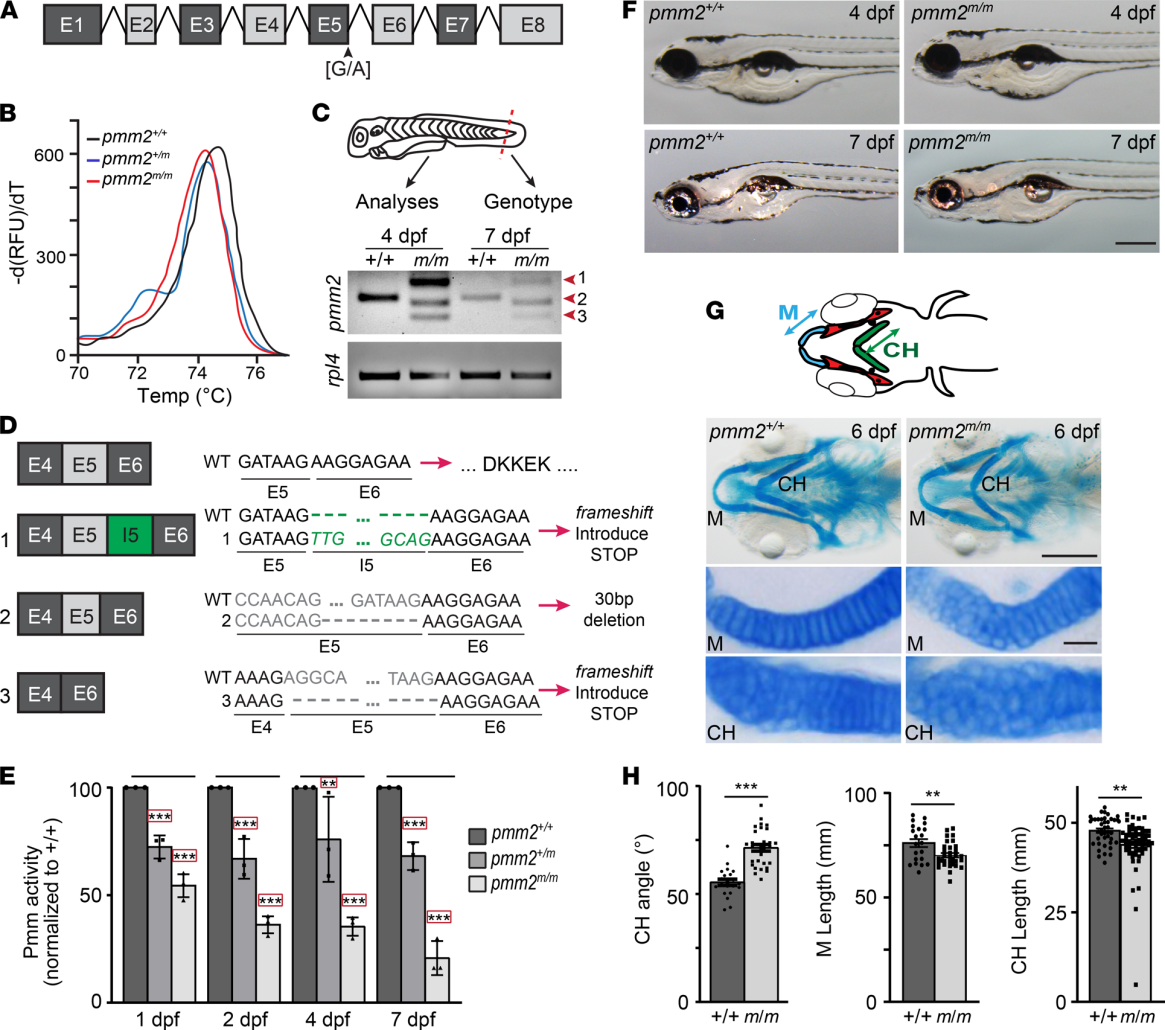
mRNA splicing is disrupted in *pmm2^m/m^* transcripts generating a hypomorphic allele. (**A**) Schematic illustrates zebrafish *pmm2*. (**B**) High resolution melting curve (HRM) analysis shows melting curves for *pmm2^+/+^*, *pmm2^+/m^* (heterozygous for the sa10150 allele), and *pmm2* [G/A] mutants homozygous for the sa10150 allele (*pmm2^m/m^*). (**C**) Schematic shows use of embryo fin for HRM genotyping. Reverse transcriptase PCR (RT-PCR) analyses reveal 3 unique *pmm2* gene products in *pmm2^m/m^* embryos (forms 1–3). (**D**) Sequencing of individual RT-PCR products shows a frameshift in forms 1 and 3 with early stop codons. Form 2 contains an in-frame truncation of exon 5, explaining the hypomorphic allele. (**E**) Pmm activity measured in embryonic lysates shows a progressive decrease in activity in *pmm2^m/m^* embryos. *n* = 3 experiments of 25 embryos per sample. Error bars show SEM, Dunnett’s test, **P < 0.01, ***P < 0.001. (**F**) Bright-field images of embryos 4 and 7 dpf show no obvious differences between *pmm2^+/+^* and *pmm2^m/m^* embryos. Scale bar: 100 μm. (**G**) Schematic illustrates several key structures of embryonic jaw, including Meckel’s cartilage (M) and the ceratohyal (CH), with arrowed lines demonstrating parameters measured. Alcian blue staining of ventral structures of 6 dpf embryos reveals differences in the shape of M and CH cartilages. Flatmount preparations show morphological alterations are associated with immature chondrocytes that are round and disorganized. *n* = 25–30 embryos per condition over 3 experiments. Scale bars: 10 μm. (**H**) Quantitation of CH cartilage angle, CH length, and M cartilage length show multiple *pmm2^+/+^* and *pmm2^m/m^* embryos. *n* = 25–30 embryos per condition over 3 experiments. Error bars show SEM, Student’s *t* test, **P < 0.01, ***P < 0.001.

The Sanger Center’s Sperm TILLING screen identified a c.152G>A (sa10150) mutation predicted to disrupt an essential splice site in exon 5 of *pmm2* (Figure 1A). F1 embryos generated by in vitro fertilization with sperm carrying the c.152G>A mutation were obtained from the Zebrafish International Resource Center. Using next-generation sequencing to analyze each of the mutations originally identified within the pool of affected sperm, we isolated multiple F1 adults carrying the *pmm2* c.152G>A variant and only 2 additional genetic alterations. Heterozygous carriers of the *pmm2* c.152G>A variant were identified using high-resolution melt curve (HRM) analyses and outcrossed with TLAB animals for more than 5 generations (Figure 1B). HRM analysis of caudal fin tissue was used to assign embryo genotype prior to all subsequent molecular and biochemical studies (Figure 1C). Matings between F5 *pmm2^+/m^* adults yielded progeny of all 3 genotypes in the expected Mendelian ratios. RT-PCR analyses indicated that while homozygous *pmm2^+/+^* embryos expressed a single transcript, *pmm2^m/m^* animals expressed 3 unique splice forms (Figure 1C). Sequencing of individual splice variants showed transcripts 1 and 3 contained shifted reading frames with early stop codons, due either to retention of intron 5 or to deletion of exon 5. In transcript 2, however, in-frame truncation of the last 30 bp of exon 5 maintained the normal reading frame and as such was predicted to produce a protein with some residual activity (Figure 1D). Alignment between human and zebrafish Pmm2 protein sequences showed that the 2 residues essential for enzyme activity (i.e., the nucleophile and the proton donor), as well as the 6 residues comprising the substrate-binding site, were conserved (Supplemental Figure 1; supplemental material available online with this article; https://doi.org/10.1172/jci.insight.153474DS1). Importantly, the 10 amino acids deleted in transcript 2 only affected 1 binding site residue and neither active site residue. Analyses of Pmm activity in *pmm2^+/+^* and *pmm2^m/m^* embryos 1–7 dpf revealed a steady decline in activity, with *pmm2^m/m^* embryos exhibiting about 55% of total activity in wild-type embryos at 1 dpf but only about 20% by 6–7 dpf (Figure 1E). This is consistent with activity levels noted in certain PMM2-CDG patient fibroblasts (typically ranging from 3% to 15% of wild-type activity) (11–13) but lower than activity levels achieved using morpholinos to inhibit enzyme expression (Supplemental Figure 1B) (9). The higher activity noted in *pmm2^m/m^* embryos 1–2 dpf likely represents maternally derived enzyme and transcript, each of which wanes by days 3–4.

### pmm2^m/m^ hypomorphs exhibit both craniofacial and motility defects.

A zebrafish line (*pmm2^it768^*) carrying the same c.152G>A mutation was reported to exhibit no phenotypic abnormalities by 5 dpf (14). Although no obvious outward abnormalities were observed in the first 5 days in the *pmm2^sa10150^* mutant, Alcian blue analyses of craniofacial cartilages revealed several defects in the lower jaws of *pmm2^m/m^* embryos (Figure 1, F–H). In particular the Meckel’s and ceratohyal cartilages were shorter and misshapen compared with wild-type structures. Flatmount preparation of dissected cartilages showed this was associated with alterations in chondrocyte morphology and organization. Unlike wild-type structures, where the majority of chondrocytes were elongated and had converged to form a single row, several regions in *pmm2^m/m^* structures contained multiple layers of immature, round cells. This is similar to the phenotypes previously noted following morpholino knockdown of *pmm2* gene expression (9).

**Figure 2:**
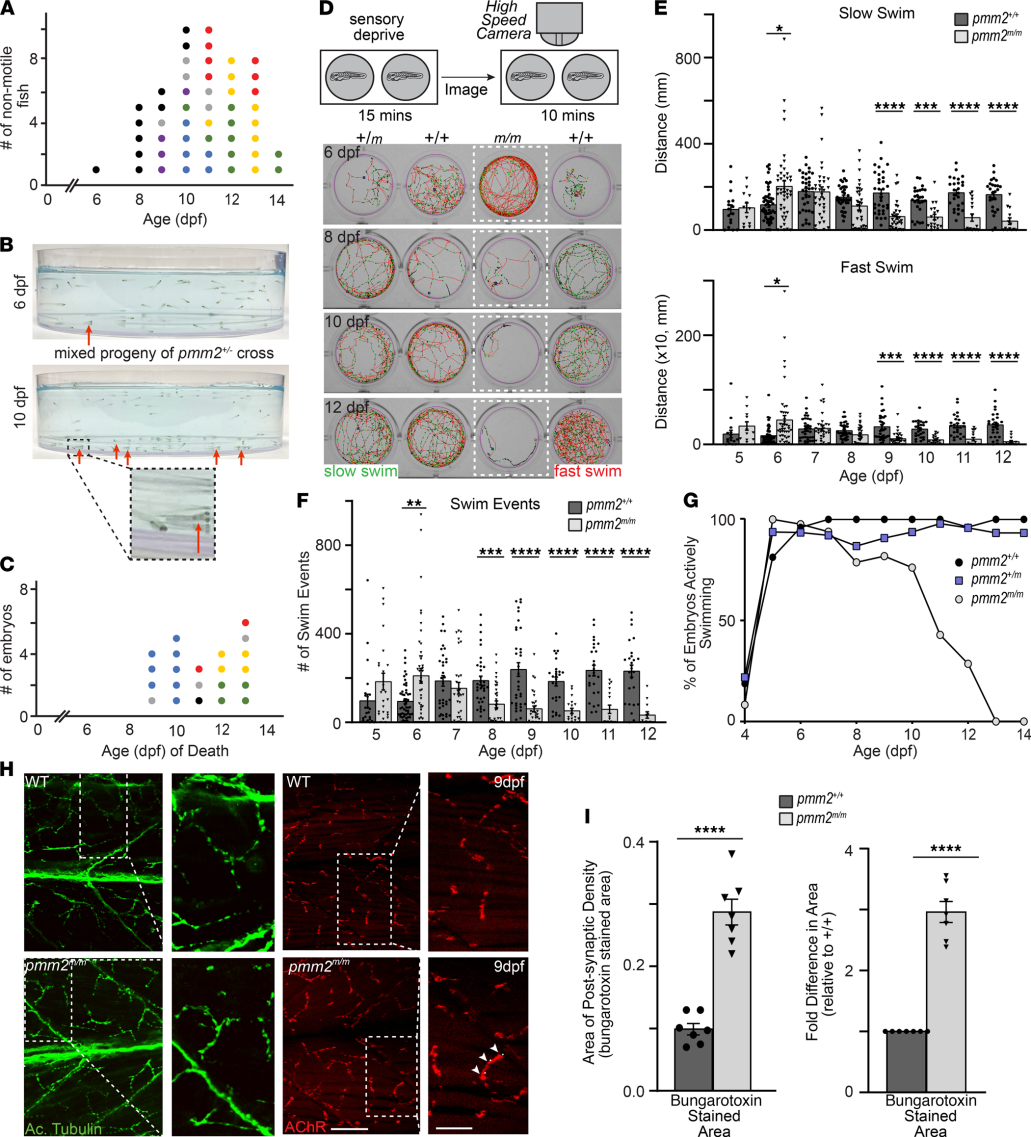
Defects in synaptic development render *pmm2^m/m^* embryos progressively nonmotile. (**A**) Eight to 14 dpf *pmm2^m/m^* embryos become progressively nonmotile. Graph shows number of nonmotile fish per day. Each colored dot represents the progeny from the same biological cross, such that all sibling animals collected on different days from that clutch are represented by the same-color dot. Genotyping showed all collected nonmotile embryos were *pmm2^m/m^*. (**B**) Lateral images of Petri dishes show *pmm2^m/m^* embryos lying on the bottom. Arrows indicate nonmotile *pmm2^m/m^* embryos. (**C**) *pmm2^m/m^* embryos die 9 to 14 dpf. Graph shows number of embryos dying each day. Each colored dot represents the progeny from the same biological cross, such that animals collected (on different days) from that clutch are siblings. (**D**) Schematic demonstrates Zebrabox behavioral analyses with 1 embryo placed per well and sensory deprived for 15 minutes. The swim paths, speeds, and general behavior are recorded for 10 minutes. Images of swim paths 6 to 12 dpf. Green paths indicate slow swim speed and red paths fast swim speeds. (**E**) Graphs show distance each embryo swam from 5–12 dpf at slow (upper) and fast (lower) swim speeds. *n* = >100 embryos per genotype over 5 experiments. Error bars show SEM, Student’s *t* test, *P < 0.05, **P < 0.01, ***P < 0.001, ****P < 0.0001. (**F**) Graph illustrates number of swim events initiated per embryo 5–12 dpf. (**G**) Graph illustrates percentage of embryos actively swimming 4–14 dpf. (**H**) Confocal images of neuromuscular systems. Motor axons are stained green with acetylated tubulin, and AChRs are stained red with bungarotoxin. Lateral images show higher power images of boxed regions. Arrowheads indicate immature, disorganized postsynaptic density. Scale bars: 20 µm and 8 µm. (**I**) Graphs show area of bungarotoxin staining. Each dot represents the average area of 15 synapses in an individual embryo. *n* = 8 embryos per condition. Error bars show SEM, Student’s *t* test, ****P < 0.0001.

In addition to the craniofacial phenotypes, *pmm2^m/m^* embryos displayed progressive motility defects, characterized by loss of both spontaneous and elicited swim behaviors. While wild-type embryos actively traversed the full depth of a Petri dish, starting 5–6 dpf, nonmotile *pmm2^m/m^* embryos were increasingly found lying on the bottom (Figure 2, A and B). Lack of movement culminated in death by 13–14 dpf (Figure 2C). Using the Zebrabox automated behavioral tracking system that utilizes a high-speed camera to document and quantitate animal motility, we further characterized these behaviors (Figure 2D). Traced paths of individual swim events revealed differences between wild-type and *pmm2^m/m^* embryos in swim speed, distance traveled, and number of swim events initiated. In particular, *pmm2^m/m^* embryos exhibited a progressive decline in the distance traveled at both fast and slow speeds, with fewer swimming events initiated (Figure 2, E and F). By 7 dpf, the number of *pmm2^m/m^* embryos that actively swam decreased rapidly, further highlighting the progressive loss of motor function (Figure 2G). Notably, *pmm2^m/m^* embryos consistently exhibited a short burst of activity, evident both in the number of swim events and total distance traveled, immediately preceding (5–6 dpf) onset of immobility. To see whether the neuromuscular system was altered in *pmm2* mutants, embryos were stained immunohistochemically with an antibody recognizing an acetylated form of tubulin, which is enriched in neurons (15), and fluorescently labeled bungarotoxin. Bungarotoxin irreversibly binds acetylcholine receptors (AChRs) in the postsynaptic portion of the neuromuscular junctions (NMJs) (16). Confocal analyses of motor axons and NMJs showed that while the axonal trajectories of *pmm2^m/m^* embryos were generally normal, the synapses present on the terminal branches of many secondary motor neurons were immature (Figure 2, H and I). By 9 dpf, this was evident as increased size of the synaptic varicosities and defects in the organization of the postsynaptic densities. Bungarotoxin-stained AChRs present in wild-type NMJs formed discrete puncta that were perfectly apposed to the presynaptic varicosity. In contrast, AChR distribution in synapses of *pmm2^m/m^* embryos was diffuse and extended beyond the bouton’s border. These findings are consistent with several synaptic phenotypes described following RNAi inhibition of *Pmm2* expression in Drosophila neurons (10). Collectively these data suggest that progressive loss of mobility exhibited by *pmm2^m/m^* embryos resulted from defective neuromuscular development, including failure to completely refine the architecture of the NMJ. It is unclear currently whether these developmental defects would eventually cause neuronal atrophy or degeneration or whether these phenotypes explain aspects of PMM2-CDG–associated ataxia.

### N-cadherin processing is reduced in pmm2^m/m^ hypomorphs.

**Figure 3:**
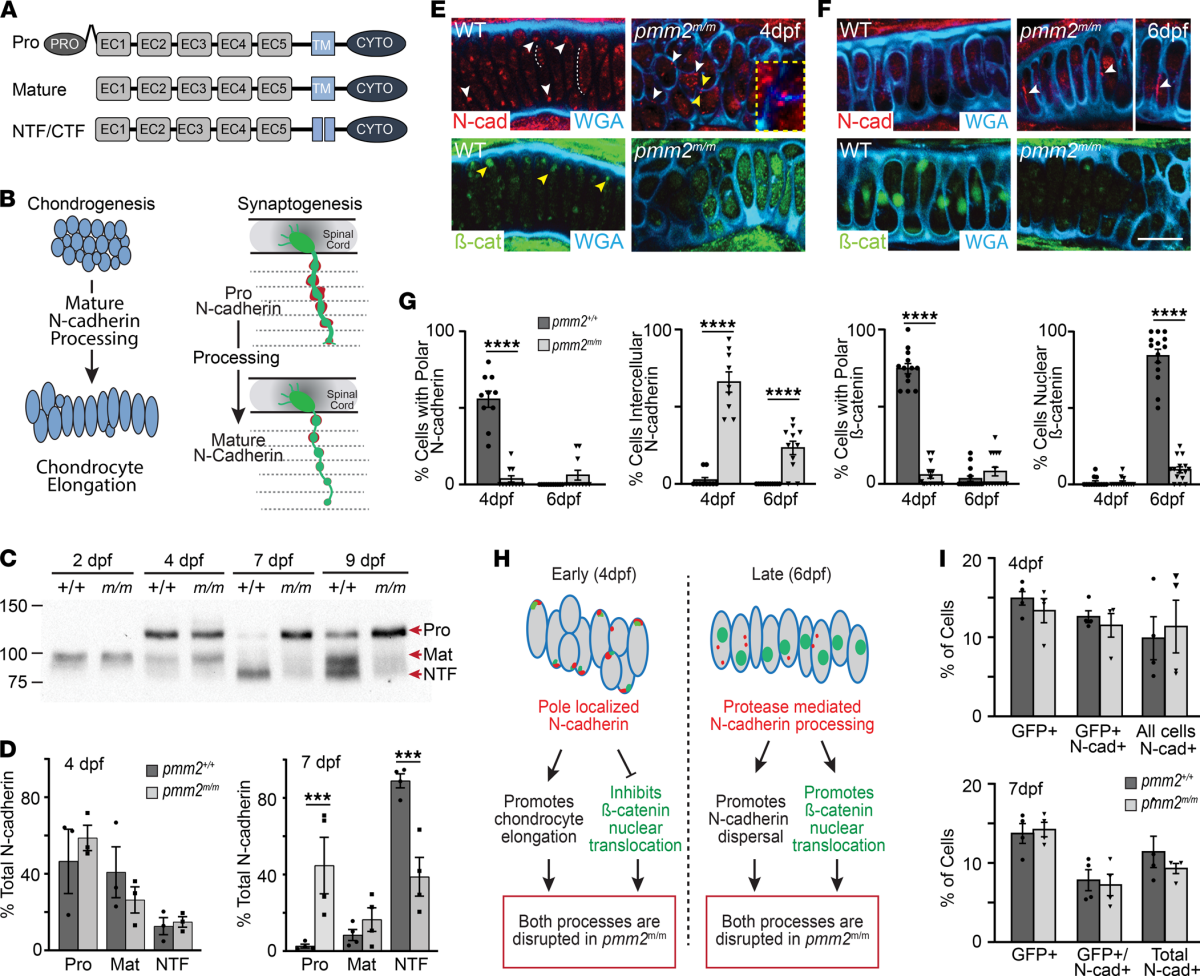
Defects in N-cadherin processing disrupt chondrogenesis in *pmm2^m/m^* embryos. (**A**) Schematic representation of N-cadherin forms. N-cadherin contains a pro domain, 5 extracellular (EC1–EC5) domains, a transmembrane domain (62), and a cytosolic (CYTO) domain. Nonadhesive pro N-cadherin is proteolytically cleaved, creating mature N-cadherin (Mat). Additional processing generates signaling-competent N- and C- terminal fragments (NTF, CTF). (**B**) Schematic illustrates role of N-cadherin forms in chondrogenesis and synaptogenesis. Axons are shown in green and bungarotoxin-stained postsynaptic densities are red. (**C**) Representative N-cadherin Western blot reveals defects in processing in *pmm2^m/m^* embryos. *n* = 3 experiments with 15 embryos per sample per experiment. (**D**) Quantification of individual protein forms. Error bars show SEM, Student’s *t* test, ***P < 0.01. (**E** and **F**) Confocal images of chondrocytes stained immunohistochemically with N-cadherin (red) or β-catenin (green). Cell surface is stained with WGA (blue). White arrows highlight N-cadherin location. In +/+ it is primarily found at the cell poles, but in *m/m* N-cadherin interactions persist on opposing cell membranes. The yellow inset is a 2.5× magnification of the original panels of N-cadherin on opposing membranes. Yellow arrows highlight β-catenin location, and white dotted line highlights N-cadherin located laterally in elongated cells. Scale bars: 10 μm. (**G**) Graphs quantitating N-cadherin and β-catenin localization. Data presented as percentage cells within an individual cartilage. *n* = 10 embryos per genotype per age over 3 experiments. Error bars show SEM, Student’s *t* test, ****P < 0.0001. (**H**) Schematic illustrates model of N-cadherin localization and processing during normal and disrupted chondrogenesis. (**I**) The level of cell surface N-cadherin present in +/+ and *m/m* embryos. Shown is the percentage of total cells that are GFP^+^, GFP^+^ and N-cadherin^+^, or N-cadherin^+^. *n* = 3 experiments of 15, with cells isolated from pools of 15 embryos per sample. Error bars show SEM.

During both chondrogenesis and synaptogenesis, the transition toward a mature cellular architecture involves fluctuations in the adhesive properties of the cell types involved. This transition is in part mediated by different forms of the cell adhesion molecule N-cadherin. N-cadherin is synthesized as a *pro* protein that is sequentially processed to first form a membrane-associated, mature molecule. This is followed by additional cleavages that create N- and C-terminal fragments (NTFs and CTFs) (Figure 3A) (17). In prechondrocytic cells, cleavage of mature N-cadherin and release of the NTF is thought to reduce intercellular adhesion and promote cell elongation (18). Studies in developing axons have further shown not only that *pro* N-cadherin is present on the cell surface but also that timed removal of the pro domain refines the size and shape of pre- and postsynaptic termini (Figure 3B) (19, 20). Western blots of wild-type and *pmm2^m/m^* embryos showed the abundance of each N-cadherin form was equivalent at early stages, but by 7 dpf *pmm2^m/m^* embryos predominantly exhibited unprocessed *pro* N-cadherin (Figure 3, C and D). This was in contrast to wild-type embryos, where little unprocessed protein was detected 7 dpf, but newly synthesized *pro* N-cadherin reappeared 9 dpf. The sudden loss of N-cadherin processing in *pmm2^m/m^* embryos corresponds with a significant drop in Pmm activity (see Figure 1E).

Confocal analyses of 4 dpf immunohistochemically stained cartilage sections showed wild-type cells contained 2 populations of N-cadherin, one present along the lateral edges of the elongated chondrocytes (Figure 3E, white dotted lines) and one present at the poles (Figure 3E, white arrows). Unlike wild-type, very little pole-localized N-cadherin was detected in *pmm2^m/m^* embryos 4 dpf; instead, it persisted at regions of cellular contact (white arrows). Since N-cadherin is linked to the cytoskeleton through its interaction with the Wnt signaling molecule β-catenin, redistributing N-cadherin to the cellular pole may contribute to cell shape changes by reorienting the cytoskeleton. In *pmm2^m/m^* embryos, its persistence at regions of cellular contact likely inhibits this process, in turn disrupting chondrocyte elongation and reorganization. The distribution of N-cadherin in wild-type chondrocytes is similar to that described in delaminating cardiomyocytes, where N-cadherin movement within the membrane promotes cardiac trabeculation (21). N-cadherin interaction with β-catenin also prevents β-catenin’s cytosolic localization and nuclear translocation, thereby regulating its stability and transcriptional activity. Immunohistochemical analyses showed β-catenin was also largely localized to the pole in wild-type cells 4 dpf (Figure 3E, yellow arrows), but predominantly cytosolic in *pmm2^m/m^* embryos. By 6–7 dpf N-cadherin staining was no longer detected in wild-type cells, and β-catenin was primarily found in the nucleus (Figure 3, F and G). In *pmm2^m/m^* embryos, however, N-cadherin persisted along cellular boundaries (Figure 3, F and G, white arrows), with very little nuclear β-catenin staining observed. These changes in N-cadherin localization and function are consistent with the transition from pro to processed forms noted in Western blot of 6–7 dpf wild-type lysates (see Figure 3C). Taken together these data suggest that under normal conditions N-cadherin processing regulated its movement from regions of cellular contact to the poles. Our data suggest these events were important for cellular elongation and β-catenin function, both of which were disrupted in *pmm2^m/m^* embryos (Figure 3H).

Although Western blot analyses suggested no obvious defects in the occupancy of N-glycans on N-cadherin, increased abundance of *pro* N-cadherin could reflect abnormally glycosylated protein retained in the endoplasmic reticulum (ER). To address this possibility, we assayed cell surface levels of N-cadherin using flow cytometry (Figure 3I). *fli1a*:EGFP-positive 4 and 7 dpf wild-type and *pmm2^m/m^* embryos were dissociated into single-cell suspensions, and the level of cell surface N-cadherin was probed. To avoid unwanted cleavage of cell surface proteins, embryos were dissociated using collagenase. No significant differences in cell surface abundance of N-cadherin were detected in either the total population or the chondrocyte-enriched, GFP-positive cells (Figure 3I and Supplemental Figure 2). As expected the level of cell surface N-cadherin declined in both wild-type and *pmm2^m/m^* cells between 4 and 7 dpf. These data are consistent with confocal analyses, which showed persistent pools of N-cadherin present on the surface of *pmm2^m/m^* chondrocytes. This suggests that although N-cadherin processing was altered, a substantial portion of the pro N-cadherin detected by Western blot in 7 dpf *pmm2^m/m^* embryos was present on the cell surface.

The cartilage phenotypes noted in *pmm2*-deficient embryos are reminiscent of alterations in Wnt planar cell polarity (PCP) and cell adhesion (22, 23), both of which are highly interwoven. To explore the extent to which alterations in N-cadherin processing and β-catenin localization affected either Wnt PCP or canonical Wnt signaling, we surveyed the expression level of several downstream targets by quantitative reverse transcriptase (qRT-PCR) (Supplemental Figure 3). For both noncanonical Wnt PCP and canonical Wnt signaling, no significant change was noted in the majority of genes assayed. Interestingly, although not always significantly altered, transcript abundance of 2 genes that also affect cellular elongation (kinesin family member 5B, a and b; *kif5Ba* and *kif5Bb*) was more dynamic at 4 dpf and 7 dpf inf *pmm2^m/m^* embryos. *kif5A* and *kif5B* loss-of-function mutants exhibit craniofacial defects that are associated with failure to maintain cellular polarity (24). These 2 kinesin genes are suggested to influence cellular polarity through mechanisms independent of either the canonical Wnt signaling or PCP pathways, instead contributing to cartilage development by transporting vesicles carrying essential matrix-remodeling molecules to the cell surface. Parallel studies using a transgenic reporter of canonical Wnt signaling also showed very little difference between wild-type andf *pmm2^m/m^* embryos in developing cartilage (not shown) (25). Additionally, Western blot analyses of global glypican abundance also showed no significant differences (Supplemental Figure 3). Together, these data suggest that although the core elements of canonical Wnt signaling and PCP were largely unaffected in *pmm2* mutants, parallel pathways regulating cell adhesion and noncanonical PCP appeared disrupted. In particular, reduced processing of N-cadherin impeded its relocalization and disrupted chondrocyte elongation. We propose N-cadherin’s absence from the cellular pole altered its interaction with β-catenin, which would normally function to reorient the actin cytoskeleton and promote cellular elongation (Figure 3H). This interaction also normally sequesters β-catenin, preventing its degradation in the cytosol, simultaneously regulating its translocation into the nucleus.

### The activities of multiple proteases, including furin PCs and Mmps, are disrupted in pmm2^m/m^.

**Figure 4:**
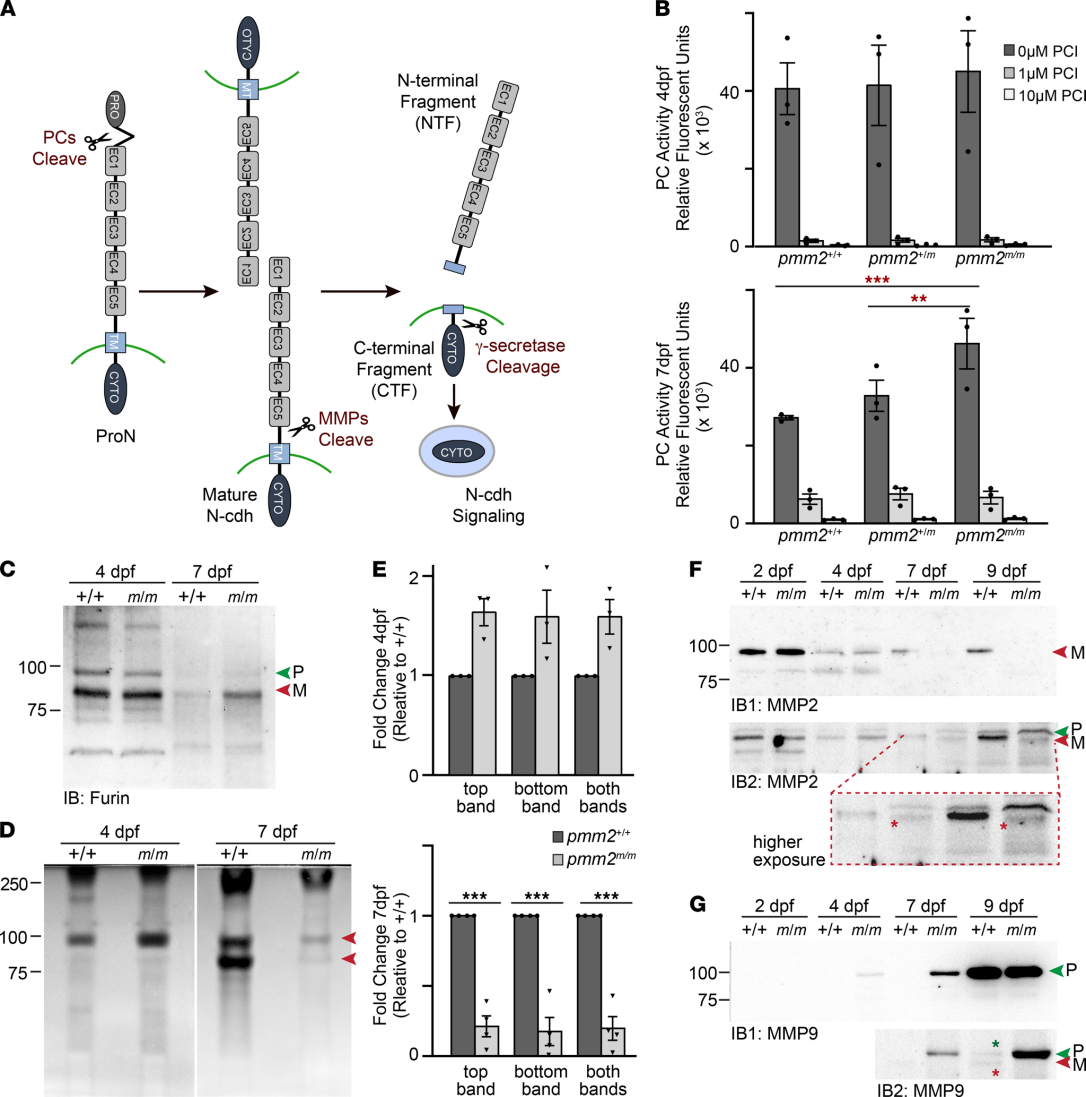
Proprotein convertase and Mmp activity is reduced in *pmm2^m/m^* embryos. (**A**) Schematic of protease-mediated N-cadherin cleavage. (**B**) In vitro enzyme assays for PCs in embryo lysates show increased activity in *pmm2^m/m^* embryos 7 dpf. *n* = 3 experiments of 15 embryos per condition per sample. Error bars show SEM, Dunnett’s test, **P < 0.01, ***P < 0.001. (**C**) Western blot of Furin enzyme in embryo lysates; P (green arrow), pro form; M (red arrow), mature form. (**D**) Gelatin zymography of embryos shows decrease in gelatinase activity in *pmm2^m/m^* embryos 7 dpf (red arrows). (**E**) Graphs quantitate gelatinase activity. *n* = 4 experiments of 15 embryos per condition per sample. Error bars show SEM, Student’s *t* test, ***P < 0.001. (**F**) Two Western blots of Mmp2. Immunoblot 2 (IB2) is shown at higher exposure with a higher magnification “inset” that illustrates the pro and mature bands. Red stars highlight a shift in Mmp2’s molecular weight in *pmm2^m/m^* embryos relative to control embryos. *n* = 3 experiments with 15 embryos per sample per experiment. (**G**) Two Western blots of Mmp9. In immunoblot 2 stars denote the pro (green star) and mature (red star) forms of Mmp9 present in *pmm2* control embryos, while only the pro form is noted in *pmm2^m/m^* embryos. *n* = 3 experiments with 15 embryos per sample per experiment.

N-cadherin processing is mediated by multiple proteases, with furin PCs removing the protein’s propeptide (Figure 4A) (26). Release of the propeptide exposes a highly adhesive N-terminal extracellular domain, which is shed when one of several Mmps cleaves outside the transmembrane domain (27, 28). Further cleavage by γ-secretase releases the intracellular CTF, itself capable of mediating N-cadherin signaling. Analysis of PC activity in embryonic lysates 4 and 7 dpf indicated that global activity was initially similar, but by 7 dpff *pmm2^m/m^* embryos contained 40% more activity than wild-type (+/+) embryos (Figure 4B). Western blot analyses of furin showed the increase in activity was associated with increased abundance of a lower molecular weight form that likely represents the mature enzyme lacking the pro domain (Figure 4C and Supplemental Figure 4A). Morpholino inhibition of furin expression in wild-type embryos demonstrated that multiple protein bands recognized by the antibody were indeed furin (Supplemental Figure 4B). It is notable that multiple processed forms were apparent in developing embryos but not typically seen in cells. It is unclear if these proteins represent processing intermediates or are in fact unique forms that are differentially presented in developing tissues.

Increased furin activity did not directly explain impaired processing of pro N-cadherin. In light of this, and of the fact that *pmm2*-deficient fruit flies exhibit less Mmp activity (10), we used a combination of gel zymography and in vitro assays to ask whether Mmps were also altered in *pmm2^m/m^* embryos (Figure 4, D and E, and not shown). While we consistently noted a slight increase in gelatinase activity in 4 dpf *pmm2^m/m^* embryos, the difference was not significant. By 7 dpf, however, the gelatinase activity detected in *pmm2^m/m^* embryos was 5-fold lower than in wild-type embryos. Using morpholinos to reduce expression of either *mmp2* or *mmp9*, we showed Mmp2 provided the majority of gelatinase activity in 7 dpf embryos, with Mmp9 minimally contributing (Supplemental Figure 4, C–E). Western blot analyses of Mmp2 also demonstrated its protein abundance was reduced in *pmm2^m/m^* embryos 7 and 9 dpf (Figure 4F and Supplemental Figure 4, F and H). Reduced abundance of the major Mmp2 band, which likely represents mature protein, was also matched with increased abundance of a higher molecular weight band (Figure 4F, higher exposure inset). These data suggest in *pmm2^m/m^* embryos reduced levels of Mmp2 activity may stem from impaired processing of the inactive pro enzyme (Supplemental Figure 4, E, F, and H). Notably, we regularly detected a small shift in the mobility of mature Mmp2. Mature Mmp2 has 2 potential N-glycosylation sites in the hemopexin domain. The reduction in molecular weight is consistent with an apparent loss of 1 to 2 N-glycans but could also reflect additional protein processing. Because Mmp2 and Mmp9 are known to influence each other’s activation (29), we also asked whether Mmp9 expression or processing was altered in *pmm2^m/m^* embryos. Surprisingly, Western blot of Mmp9 showed increased abundance in *pmm2^m/m^* embryos (Figure 4G and Supplemental Figure 4, E–H). Based on its electrophoretic mobility, morpholino-mediated depletion, and the observation that Mmp9 does not appear to contribute significant gelatinase activity at 7 dpf (Supplemental Figure 4, D and E), we believe the protein detected in *pmm2* mutants represents the inactive pro enzyme. As shown in a higher exposure inset of another experiment, low levels of both pro and mature Mmp9 could be detected in wild-type embryos (Figure 4G, higher exposure of second blot). Comparison of these forms confirmed that levels of pro Mmp9 were increased in *pmm2^m/m^* embryos. qRT-PCR analyses of several protein PCs, as well as *mmp2* and *mmp9*, showed decreased transcript abundance of 4 major PCs (*furina*, *furinb*, *pcsk5a*, and *pcsk5b*) with little change in the abundance of either *mmp2* or *mmp9* (Supplemental Figure 4J). Importantly, however, *mmp9* transcript abundance was very low in both wild-type and *pmm2^m/m^* embryos. Together, these data suggest the activities of multiple proteases, including furin and Mmp2 and Mmp9, are disrupted in *pmm2* mutants. For each enzyme, altered activity is associated with aberrant processing, with Mmp2 and Mmp9 persisting in their inactive pro forms and furin more prevalent in the active mature form.

### Inhibition of furin PCs rescues multiple molecular and cellular phenotypes.

**Figure 5:**
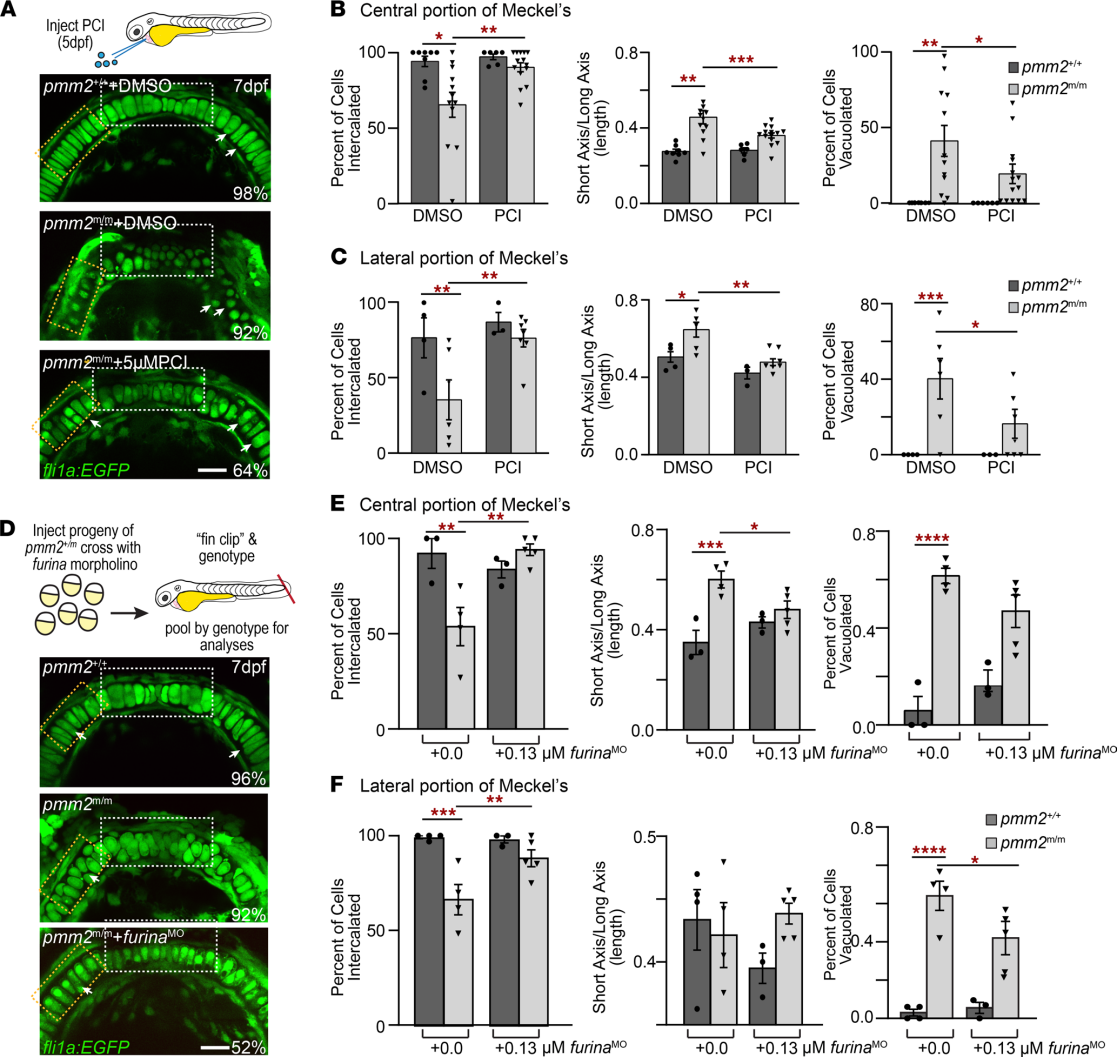
PC inhibition improves craniofacial phenotypes in *pmm2^m/m^* embryos. (**A**) Pericardial injection of PC inhibitor. Confocal images of *fli1a*:EGFP-labeled cartilage structures show PCI injection improves chondrocyte morphology, organization, and differentiation in *pmm2^m/m^* embryos. White boxes, central portion of Meckel’s cartilage; orange boxes, lateral regions evaluated for phenotypic rescue. Scale bars: 10 μm. Percentage values indicate number of scored embryos exhibiting pictured phenotype. *n* = 30 embryos over 4 experiments. (**B**) Parameters assessed for rescue of central portion of Meckel’s cartilage (white boxes). Cells were scored for percentage of intercalated, ratio between long and short axis (measure of roundness), and percentage of cells with vacuoles. *n* = 8–15 treated embryos over 4 experiments. (**C**) Parameters assessed for rescue of lateral portion of Meckel’s cartilage (white boxes). Cells were scored as in **B**. *n* = 8–15 treated embryos over 4 experiments. (**D**) Experimental strategy involving injecting a morpholino targeting *furina* into mixed progeny of *pmm2^m/m^* incross at 1-cell stage and genotyping 3 dpf. Confocal images of *fli1a*:EGFP-labeled cartilage structures show inhibiting furina improves chondrocyte morphology, organization, and differentiation in *pmm2^m/m^* embryos. White boxes, central portion of Meckel’s cartilage; orange boxes, lateral regions evaluated for phenotypic rescue. Scale bars: 10 μm. Percentage values indicate the number of scored embryos exhibiting pictured phenotype. *n* = 8–15 treated embryos over 4 experiments. (**E**) Parameters assessed for rescue of central portion of Meckel’s cartilage (white boxes) following morpholino inhibition of *furina*. Cells were scored as in **B**. (**F**) Parameters assessed for rescue of lateral portion of Meckel’s cartilage (orange boxes) following morpholino inhibition of *furina*. Cells were scored as in **B**. Error bars show SEM, 2-way ANOVA, *P < 0.05, **P < 0.01, ***P < 0.0001, ****P < 0.0001.

Knowing that furin PCs can directly and indirectly influence Mmp activation (30–32), we asked whether inhibiting their activity improved the molecular and cellular phenotypes associated with *pmm2* deficiency. Remarkably, injection of a pan-reactive proconvertase inhibitor (PCI) into the hearts of 5 dpf *pmm2^m/m^* embryos significantly improved cartilage morphology in 64% of the embryos analyzed (Figure 5, A–C). Phenotypic rescue was assessed 7 dpf in the central and lateral portions of Meckel’s cartilage using multiple parameters, including cellular shape, organization, and degree of vacuolation. Cells present as a single row were considered fully intercalated, while those found in a multilayered configuration were not. PCI treatment increased cellular intercalation in both regions of Meckel’s cartilage in *pmm2^m/m^* embryos by 28%. Similarly, cell shape, which was quantitatively measured by calculating the ratio between the short and long cellular axes, was also significantly improved in both regions following PCI treatment. In mutant embryos, increased cellular roundness resulted in a much larger ratiometric value that was reduced 22% when PC activity was inhibited (Figure 5C). Finally, a large percentage of the chondrocytes in the lateral and central regions of *pmm2^m/m^* cartilage contained visible vacuoles that also disappeared when PC activity was inhibited. Together, these data suggest that enhanced activity of at least 1 PC enzyme expressed in developing embryos plays a central role in *pmm2^m/m^* cartilage pathology. To ask whether furin, the prototypical proprotein convertase, contributes to *pmm2* pathogenesis, we used morpholinos to genetically reduce expression of *furina*, whose disruption impairs craniofacial cartilage development (33). As noted with pharmacological inhibition, reducing *furina* expression also improved cartilage dysmorphia in 53% of *pmm2^m/m^* embryos (Figure 5, D–F). These data suggest that alterations in the activity of PCs, like furin, initiates a cascade of events that cause cartilage disease in PMM2-CDG.

**Figure 6:**
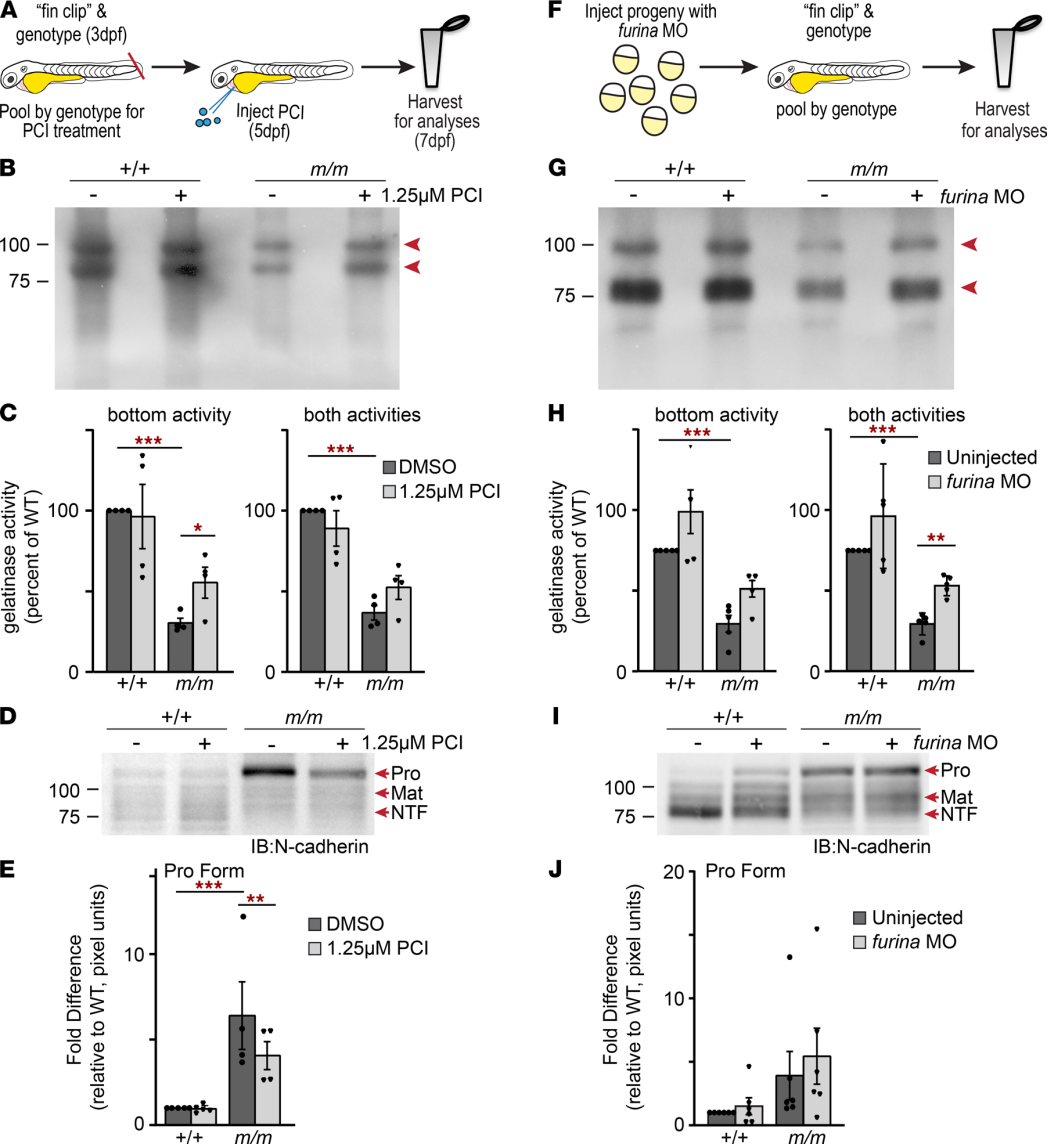
PC inhibition rescues molecular and cellular phenotypes. (**A**) Schematic shows experimental workflow. (**B**) In-gel zymography shows reducing PCs improves gelatinase/Mmp activity in *pmm2^m/m^* embryos. (**C**) Graph illustrates densitometry-based quantitation of gelatinase activity. *n* = 4 experiments, each with 15 embryos per sample per experiment. Error bars show SEM, 2-way ANOVA, *P < 0.05, ***P < 0.001. (**D**) Representative Western blot of N-cadherin in embryonic lysates with and without PCI treatment. (**E**) Graph of densitometry-based quantitation of pro N-cadherin abundance. *n* = 4 experiments, each with 15 embryos per sample per experiment. Error bars show SEM, 2-way ANOVA, **P < 0.01, ***P < 0.001. (**F**) Schematic illustrates workflow of *furina* morpholino injection, genotyping, and analyses. (**G**) In-gel zymography shows reducing *furina* expression improves gelatinase/Mmp activity in *pmm2^m/m^* embryos. (**H**) Graph demonstrates densitometry-based quantitation of gelatinase. *n* = 4 experiments, each with 15 embryos per sample per experiment. Error bars show SEM, 2-way ANOVA, **P < 0.01, ***P < 0.001. (**I**) Representative Western blot of N-cadherin in embryonic lysates shows treatment does not improve abundance of the pro form in *pmm2^m/m^* embryos. *n* = 4 experiments, each with 15 embryos per sample per experiment. (**J**) Graph of densitometry-based quantitation of pro N-cadherin. Error bars show SEM.

Consistent with this, pharmacological and genetic inhibition of PCs also increased Mmp activity and improved N-cadherin processing in *pmm2^m/m^* embryos. By 7 dpf, embryos that were injected with a PCI at 5 dpf exhibited a 34% increase in Mmp activity (Figure 6, A–C). This was accompanied by a 37% reduction in the abundance of pro N-cadherin (Figure 6, D and E). Similarly, morpholino inhibition of *furina* also improved Mmp activity in *pmm2^m/m^* embryos (Figure 6, F–H), but it had little effect on N-cadherin processing (Figure 6, I and J). Given the fact that morpholino knockdown of *furina* did restore cartilage pathology in 53% of the embryos analyzed, failure to improve processing may reflect the morpholino’s waning impact. Because morpholino injection inhibits Furina activity starting at 0 hours post fertilization and N-cadherin processing was assessed 7 dpf, we suspect the effect of *furina* knockdown had subsided. Alternatively, these data may indicate that multiple protein PCs are disrupted in *pmm2^m/m^* mutants, with one impairing Mmp activity and another N-cadherin processing. Collectively, these findings support a hypothesis where protein PCs like Furin influence the activation and activity of Mmps in developing cartilage. The data further suggest the coordinated action of PCs and Mmps further regulates processing and activity of N-cadherin, with disruptions in N-glycosylation impairing this process (Supplemental Figure 4K).

### Sulforaphane treatment does not improve cartilage phenotypes.

Previous studies in an independent zebrafish line carrying the same *pmm2^it768^* (C.152G>A) variant showed increased expression of several genes in the ER stress and unfolded protein response pathways in *pmm2*-deficient livers (14). To see whether increased ER stress contributes to cartilage phenotypes in *pmm2^sa10150^*, we performed qRT-PCR analyses of multiple genes, followed by in situ hybridization to assess cartilage-specific expression of binding immunoglobulin protein (*bip)* and C/EBP homologous protein (*chop*) (Supplemental Figure 5, A and B). While very little difference was noted in transcript abundance of *canx* or *gadd45* at either 4 or 7 dpf, *bip* and *chop* transcript levels were increased in *pmm2^m/m^* embryos at both time points. Increased transcript abundance was particularly prominent in the livers and craniofacial cartilages. Increased liver expression was previously noted by Mukaigasa et al., who subsequently explored whether stimulating NF-E2 related factor 2–dependent (*nrf2*-dependent) proteostasis improves phenotypes (14). To similarly ask whether increasing protein turnover reduces ER stress and improves phenotypes, embryos were treated starting 3 dpf with 45 μM sulforaphane. Sulforaphane is a phytochemical highly enriched in cruciferous vegetables that has been shown to stimulate *nrf2*-dependent protein turnover (34). Although sulforaphane treatment substantially reduced transcript abundance of both *bip* and *chop*, no phenotypic amelioration was noted (Supplemental Figure 5, C–E). In fact 33% of the *pmm2^m/m^* embryos treated with sulforaphane exhibited more severe cartilage dysmorphia than DMSO-treated embryos. Further sulforaphane treatment and the associated reduction in *bip* and *chop* expression exacerbated reductions in Mmp activity and N-cadherin processing (Supplemental Figure 5, F–I). Our data suggest that *pmm2^m/m^* cartilage phenotypes did not result from ER stress. In fact, increased expression of proteins like *bip* and *chop* that function to improve protein folding may protect embryos from more severe disease. It is also notable that Mukaigasa et al. did not find increased ER stress or defects in craniofacial cartilage in the *pmm2^it768^* mutants (14). Because these animals carry the same genetic variant, it is plausible that differences in genetic background mitigated onset of the cartilage phenotypes.

### LLO abundance is not appreciably altered in pmm2^m/m^ embryos, but matrix-assisted laser desorption ionization mass spectrometry imaging shows alterations in released N-glycan profiles.

Despite some indication of altered mobility of Mmp and furin proteases, it is currently unclear whether altered glycosylation directly or indirectly disrupts the function of individual enzymes. Therefore, to address whether global glycosylation is altered in *pmm2^m/m^* embryos, we used fluorophore-assisted carbohydrate electrophoresis to analyze steady-state levels of LLOs, free glycans, and sugar phosphates (35, 36). These experiments did not reveal any robust decreases in full-length LLO or increases in LLO assembly intermediates (Supplemental Figure 6), any appreciable alterations in free glycan pools, or any obvious differences in sugar phosphate levels (not shown).

**Figure 7:**
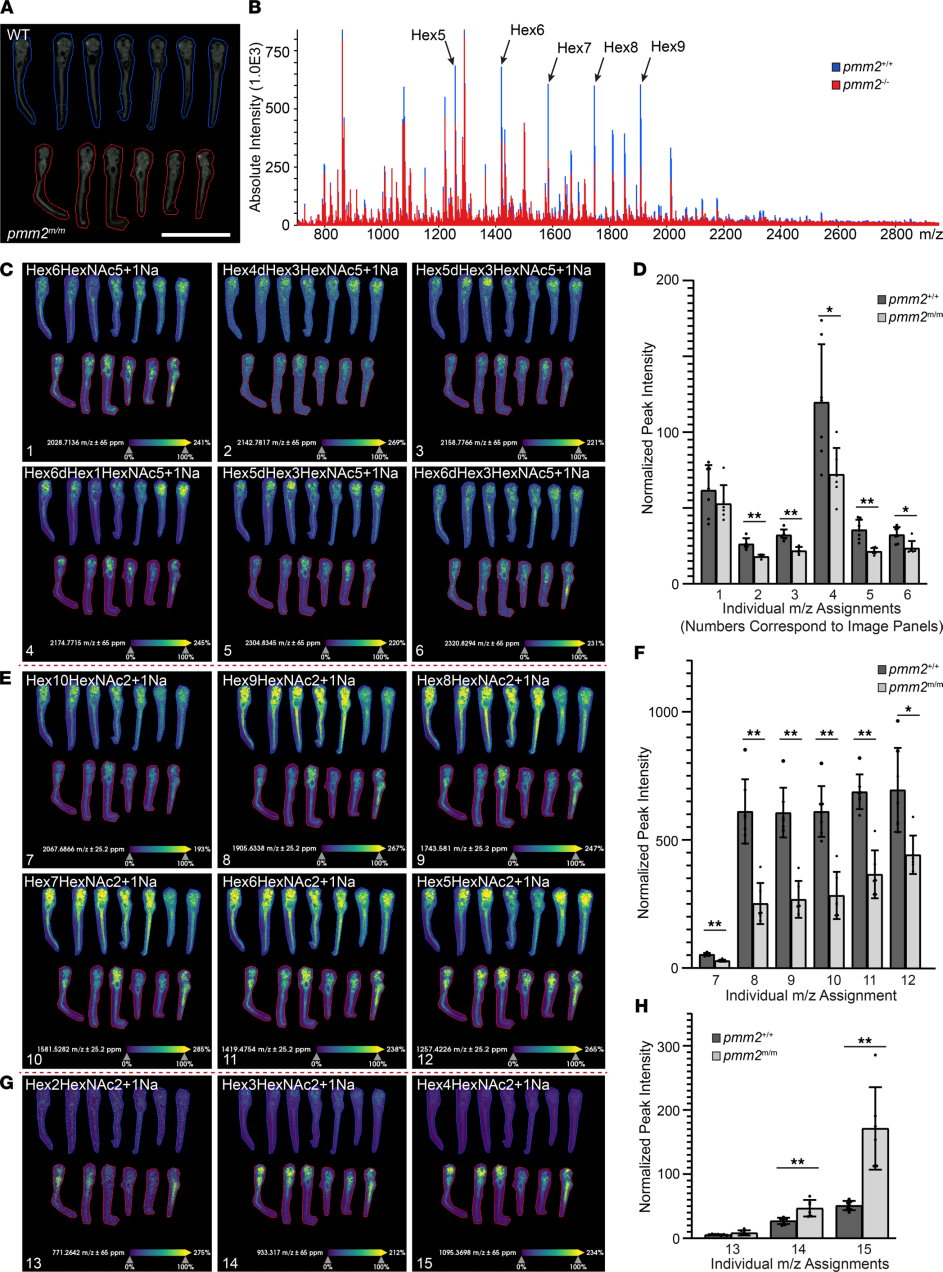
MALDI MS imaging reveals specific defects in N-glycosylation. (**A**) Representative image of embryo sections analyzed by MALDI MS imaging. Individual areas imaged are outlined in red (*pmm2^m/m^*; *n* = 5) or blue (*pmm2+/+*; *n* = 7). Scale bar: 2 mm. (**B**) Overall average spectral comparison of *pmm2^+/+^* and *pmm2^m/m^* demonstrates changes in N-glycosylation. Select contrasting high-mannose [Hex(*n*)] peaks are highlighted with arrows. (**C** and **D**) Representative images of complex-type N-glycans. Numbers on image panels correspond to bars on graph. Graph quantifying differences in complex glycans between *pmm2^+/+^* and *pmm2^m/m^* embryos. Each dot represents quantification of peak intensity from a single embryo. Data represent mass to charge ratio (*m/z*) indicative of glycan identity. Error bar shows standard deviation, Mann-Whitney *U* test, *P* < 0.01 considered significant, *P < 0.01, **P < 0.001. (**E** and **F**) Representative images of high-mannose N-linked oligosaccharides. Numbers on image panels correspond to bars on graph. Graph quantifying differences in high-mannose N-linked oligosaccharides between *pmm2^+/+^* and *pmm2^m/m^* embryos. Each dot represents quantification of peak intensity from a single embryo. Data represent *m/z* indicative of glycan identity. Error bar shows standard deviation, Mann-Whitney *U* test, *P < 0.01, **P < 0.001. (**G** and **H**) Representative images of truncated N-glycans. Graph quantifying differences in truncated sugars between *pmm2^+/+^* and *pmm2^m/m^* embryos. Each dot represents quantification of peak intensity from a single embryo. Error bar shows standard deviation, Mann-Whitney *U* test, *P* < 0.01 considered significant, **P < 0.001.

To further investigate possible differences in protein-bound N-glycans, parallel studies using more sensitive matrix-assisted laser desorption ionization mass spectrometry–based (MALDI MS–based) imaging of wild-type and *pmm2^m/m^* embryo sections were performed. This method relies on release of N-linked glycans using the endoglycosidase PNGase F followed by mass spectrometric determination of the mass to charge ratio (*m/z*) of the released glycan in a spatial manner (37, 38). These data show N-glycans are abundant throughout 6 dpf embryos, with the highest concentration noted in the head region (Figure 7). Using this more sensitive assay, we found levels of complex-type N-glycans were reduced 27%–40% in *pmm2^m/m^* embryos compared with wild-type embryos. These data suggest that while complex N-glycans’ abundance was reduced, a substantial level of N-glycan processing was preserved in the mutant embryos (Figure 7, C and D). Notably, we also detected a modestly larger reduction (37%–59%) in the abundance of the larger unprocessed, high-mannose-type N-glycans in the *pmm2^m/m^* embryo (Figure 7, E and F). This reduction was associated with an increase in the smaller unprocessed N-glycans (Figure 7, G and H). These latter structures were only minimally detected in the wild-type embryos but widely prevalent in the mutants. Similar small N-glycans have also been noted in the sera, plasma, and skin fibroblasts from patients with PMM2-CDG, suggesting a common pathomechanism for generation of this biomarker (39). Together these data indicate that certain glycoproteins are either hypoglycosylated or erroneously glycosylated with truncated oligos in PMM2-CDG, with the effects on high-mannose intermediates being more substantial than those on more processed N-glycans. It is important to note, however, that the degree to which a particular pool of N-glycans is altered does not necessarily reflect its relative contribution to resulting phenotypes.

## Discussion

Altered glycan biosynthesis and processing are associated with many common human disorders, including cancer, diabetes, and cardiovascular disease. The evidence for disease- and tissue-specific changes in glycosylation in these conditions is abundant, and in several cases alterations in glycan structure have been linked to the impaired function of specific glycoproteins (40–43). Similar evidence explaining the pathogenesis of CDG is mostly lacking. Defining glycosylation-related disease mechanisms remains challenging for CDG in part because many of the accessible CDG cell types and animal models have not revealed robust glycosylation defects on specific glycoproteins. This is particularly true for CDG that influence the biosynthesis of N-glycan precursors and N-glycan occupancy. Identifying sensitive glycoproteins in the context of these disorders is often compared to finding a needle in a haystack. Nonetheless, it is appreciated that loss of 1 or more N-glycans on a single glycoprotein is sufficient to compromise cell function and tissue development. Here, we provide new insight into the pathogenesis of PMM2-CDG by identifying protease-dependent defects in N-cadherin processing that contribute to the cartilage phenotypes in a potentially new zebrafish model. Through biochemical analyses and developmental phenotyping of *pmm2* mutants, we show defects in N-cadherin processing stem from increased activity of furin PCs. Consistent with previous studies in Drosophila, increased furin activity was associated with reduced processing and activation of Mmps 2 and 9, such that inhibiting furin PCs restored Mmp activity, N-cadherin processing, and chondrocyte morphology in *pmm2* mutants (summarized in Supplemental Figure 4K). The implications of these findings with regard to the developmental pathogenesis of PMM2-CDG are discussed.

Although N-cadherin processing and maturation were reduced in *pmm2*-mutant embryos, Western blot analyses suggested their N-glycan occupancy may be unaffected. However, both the occupancy and form of site-specific glycans have been shown to play a role in localization, processing, and dimer formation of both N- and E-cadherin (44–46). Given the low resolution of Western blot analyses for glycosylation status, we cannot currently rule out the possibility that N-cadherin’s glycosylation status was affected in *pmm2* mutants. Despite this, our data currently suggest that the primary underglycosylated proteins encompass the subset of proteases, including the furin PCs and Mmps, responsible for N-cadherin processing. The activities of PCs and Mmps are both clearly abnormal, but our present data do not definitively demonstrate loss of site occupancy on these enzymes. We did observe a shift in the electrophoretic mobility of Mmp2 that is consistent with a possible reduction in occupancy of 1–2 glycans but cannot currently rule out other possibilities. This phenotype also corresponds to a transition toward unprocessed pro Mmp2. While it remains unclear how N-glycan occupancy on each of Mmp2’s 2 potential sites regulates activation in vivo, the fact they are located within the hemopexin domain suggests they likely influence protein interactions and propeptide removal. Similarly, the single N-glycan present in the pro domain of Mmp9 is thought to modulate its activation (47–49). Future experiments aimed at defining in vivo how the glycosylation status of these enzymes impacts their activity and interactions are essential but will require generating transgenic tools that facilitate biochemical analyses in *pmm2*-mutant embryos. Given the number of cell surface and secreted proteins PCs and Mmps modify, their disruption likely also affects other cell surface proteins in *pmm2* mutants.

The selective defects in N-glycosylation we identified in *pmm2* mutants were further supported by analysis of N-glycosylation precursors, which did not show appreciable effects at steady state. Specifically we did not detect statistically significant decreases in LLO abundance. High-resolution MALDI MS imaging of released N-glycans in sections of 6 dpf *pmm2*-mutant embryos did, however, show interesting differences in the abundance of certain released N-linked glycans. Most strikingly, several truncated N-glycan structures accumulated in the PMM2-CDG embryos. These structures were present at very low levels in WT embryos, suggesting they represented abnormal processing events or accelerated degradation of incomplete N-glycans. Conversely, the zebrafish oligosaccharyltransferase complex may be capable of transferring incomplete structures to nascent polypeptides. MALDI MS imaging also revealed a 37%–59% decrease in abundance of several high-mannose structures in *pmm2*-mutant embryos. Despite this, the abundance of complex N-glycans was largely preserved, with most forms decreased 27%–40%. These data suggest that the abnormal processing and/or reduction of high-mannose structures does not cause a complete global loss of most mature N-glycans in *pmm2*-deficient embryos. Further, although reduced levels of high-mannose structures were noted throughout the embryo, alterations in complex N-glycans and paucimannose structures were concentrated in the head and craniofacial tissues. It is unclear to what degree the molecular and cellular phenotypes described stemmed from reduced levels of complex or high-mannose-type N-glycans versus the presence of truncated structures. We hypothesize that the level of hypoglycosylation or erroneous addition of truncated structures in these embryos is sufficient to influence select sensitive targets, like furin or Mmp2, but not significant enough to overwhelm the ER-associated quality control mechanisms that maintain proteostasis. In support of this hypothesis, the transcript abundance of *bip* and *chop*, 2 genes involved in the ER stress pathway, was increased in *pmm2* mutants. Reducing their expression with sulforaphane exacerbated *pmm2*-mutant phenotypes, suggesting that enhanced quality control allowed the developing embryo to maintain normal site occupancy on most N-glycoproteins despite decreased abundance of early precursors. This mechanism likely protected the embryo from more severe disease.

Analyses of *pmm2^m/m^* embryos showed certain proteolytic enzymes, including the PCs and Mmps, were particularly sensitive to hypoglycosylation. A role for Mmps in PMM2-CDG pathogenesis is suggested from studies in *pmm2*-deficient Drosophila (10), where siRNA-mediated inhibition of *pmm2* expression disrupts formation of neuromuscular synapses. Histological analyses indicate that reduced Wnt signaling in synapses of Pmm2-deficient fruit flies may relate to altered glycosylation of Mmp2, which in Drosophila is a membrane type Mmp (10). Here we definitively demonstrate reduced activity of Mmp2 (and possibly also Mmp9) stemming from failure to process the inactive pro enzyme. In vertebrates Mmp2 and Mmp9 are soluble secreted enzymes, with MT1-Mmp (Mmp14) being the prototypical membrane type enzyme. Our data suggest that reductions in Mmp activity are linked to increased activation of a furin PC, and together these activities influence N-cadherin processing. Inhibition of PCs increased Mmp activity and restored normal levels of pro N-cadherin, supporting this mechanism. How PCs regulate Mmp and N-cadherin processing remains unclear. However, MT1-Mmp, the major activator of pro-Mmp2, is itself activated at the cell surface by furin (50). Furthermore, cell surface interaction between N-cadherin and one of several membrane type Mmps, including MT1-Mmp, is thought to facilitate N-cadherin’s processing. This may occur through the ability of MT-Mmps to function as a scaffold. Reduced glycosylation on 1 or more of these Mmps could disrupt its localization and alter activation.

In addition to alterations in PC and Mmp activity, the craniofacial and synaptic defects in *pmm2* mutants were consistent with alterations in cell adhesion pathways and noncanonical Wnt PCP. Although we did not detect substantial differences in transcript abundance of genes in either pathway, the global abundance of several targets was more variable in *pmm2^m/m^* embryos. These data suggest the pathways may be locally altered in certain tissues. Among these the transcript levels of *kif5Ba* and *kif5Bb* were consistently altered. Kif5B traffics MT1-Mmp to the cell surface, and *kif5Bb* loss-of-function mutants exhibit craniofacial defects that are associated with failure to maintain cellular polarity (24, 51, 52). This is strikingly similar to the phenotypes described here in *pmm2^m/m^* embryos and also consistent with the craniofacial and synaptic defects noted in our previously characterized morphant model. It is unclear why another zebrafish line (*pmm2^it768^*) carrying the same c.152G>A mutation was reported to exhibit no overt phenotypic abnormalities in the first 5 dpf (14). The authors of this study suggest that residual activity levels may limit onset of phenotypes, but they also demonstrate significant decreases in N-glycan precursors. More likely, differences in the genetic background may account for the variable phenotypes between the *pmm2^it768^* and *pmm2^m/m^* mutants. Our finding that disrupted activity of furin PCs and Mmps was associated with altered processing of N-cadherin is a significant advance in our understanding of PMM2-CDG pathogenesis. These data highlight proteases as pathogenic drivers and provide an important platform for future studies focused on linking these phenotypes to specific defects in N-glycosylation.

## Methods

### Zebrafish strains, maintenance, and husbandry.

Animals were maintained according to standard protocols. The following zebrafish strains were obtained from the Zebrafish International Resource Center (ZIRC): TL, AB, *Tg(fli1a:EGFP)^y1^* (53), and *pmm2^sa10150^* containing a *pmm2* point mutation. Staging was done according to established criteria (54). In some cases, 0.003% 1-phenyl-2-thiourea was added to embryo medium to block pigmentation.

### Identification of the pmm2 mutation.

Primers were designed for detection of the *pmm2* sa10150 mutation and the known background mutations of the sa10150 line: *eif2a*, *hic1*, *ilvbl*, *leng8*, *magi3a*, *nod2*, *ptprja*, and *trpc5*. Sequences for all primers used are listed in Supplemental Table 1. Genomic DNA (gDNA) was extracted from embryos or fin tissue using the Extracta DNA Prep for PCR-Tissue kit (catalog 95091-025, Quanta Biosciences). HRM analyses were done using Accumelt HRM SuperMix (catalog 95103-012, Quanta Biosciences). *Pmm2^+/m^* animals were outcrossed with TLAB 5 generations prior to analyses. No known background mutations were detected after F3.

### Reverse transcriptase PCR and qRT-PCR analyses.

Primer pairs were validated for specificity and efficiency prior to use. Sequences for all primers used are listed in Supplemental Table 1. Total RNA isolation of single embryos was done using TRIzol Reagent (Ambion, Thermo Fisher Scientific) according to manufacturer’s instructions with an additional DNAse digestion added to remove contaminating gDNA. cDNA was synthesized using the qScript cDNA synthesis kit with 500 ng RNA input (95048-025, Quanta Biosciences). gDNA contamination was checked in control samples lacking reverse transcription. Ribosomal protein L4 was used as a normalization control as described previously (55). qRT-PCRs were performed using PerfeCTa SYBR Green FastMix (catalog 95072-250 Quanta Biosciences) on a Bio-Rad CFX96 Real Time System (C1000), and data analysis was performed using the CFX Maestro 1.1 (version 4.1.2433.1219) software.

### Morpholinos and inhibition of gene expression.

Morpholino knockdown of *pmm2* was performed and assessed as previously described (9). Knockdown of *mmp9* was performed with a previously described morpholino (56). For knockdown of *mmp2*, a morpholino targeting the 5′-UTR and start codon (AUG; 5′-AAAACTTAACGGACATCATGCTGGT-3′), was designed (Gene Tools). Knockdown of *furina* was performed with a previously described morpholino (33). Knockdown of *mmp2*, *mmp9*, and *furina* was confirmed by Western blot analysis.

### Pmm activity assay.

Progeny of *pmm2^+/m^* incrosses were individually fin clipped and genotyped by HRM. For embryos 1–4 dpf, the yolks were manually removed and embryos combined by genotype. Pmm activity assay was performed as previously described (9).

### Histochemistry, immunohistochemistry, and whole mount in situ analysis.

Alcian blue staining was performed as previously described (57). Stained animals were photographed on an Olympus SZ16 stereoscope outfitted with an Olympus DP73 camera. Cartilage structures were measured using CellSens software (Olympus). To account for differences in embryonic size, all measurements were normalized to the distance between the eyes. For immunohistochemical stains, the primary antibodies used included anti–acetylated tubulin (T6793, MilliporeSigma), anti–N-cadherin (ab211126, Abcam), and anti–β-catenin (PLA0230, MilliporeSigma). Whole mount immunohistochemistry was performed as described previously (57). Confocal images were acquired on an Olympus FV3000 laser-scanning microscope and images subsequently processed using both NIH ImageJ (Java 64-bit 1.52K) and Adobe Photoshop (CS6, Version 13.0). Whole mount in situ hybridizations were performed as previously described (58). In situ probes for *bip* and *chop* were generated using expressed sequence tag plasmid templates (CB865, acquired from ZIRC, and 7157773, acquired from Horizon Biodiscovery) to generate PCR products containing a T7 promoter. Primers used are listed in Supplemental Table 1.

### Zebrabox motility assay.

Larvae were placed 1 per well into 12-well plates (Cellstar, Greiner-Bio One) containing 2 mL embryo medium. Locomotor activity was monitored from 5 to 12 dpf using the Zebrabox System (ViewPoint Inc.). For analyses plates were placed into the sound deprivation chamber (part of the Zebrabox system) and desensitized for 15 minutes, with behavior subsequently recorded for 10-minute intervals. The low detection threshold was set to 20. The large activity threshold was set to 8, and the inactive threshold was set to 4. Data were analyzed using GraphPad Prism software (Version 8.1.0).

### SDS-PAGE, Western blotting, and zymography.

Embryos were manually deyolked and harvested at time points indicated. For Western blot sample preparation, 1 hour of lysis was done using 100 mM Tris pH 7.5 buffer containing 2% SDS, 2% Triton X-100, and protease inhibitor cocktail (Pierce, Thermo Fisher Scientific). Lysates were homogenized by probe sonication and centrifuged at 14,000*g* and 4°C 4°C. Protein concentrations were determined by BCA assay (Pierce, Thermo Fisher Scientific), and equal amounts of protein were loaded on SDS-PAGE gels. Blots were probed using an N-cadherin antibody (1:1000, catalog ab211126; Abcam), an MMP2 antibody (1:750, catalog AF902; R&D Systems, Bio-Techne), an MMP9 antibody (1:500, catalog AS-55345; AnaSpec), or a Furin antibody (1:2500, catalog AF1503; R&D Systems, Bio-Techne). Appropriate HRP-labeled secondary antibodies (GE Healthcare, now Cytiva; catalog 711-036-152 and 705-036-147) were used and blots analyzed using the Bio-Rad MP ChemiDoc system. For zebrafish zymography, embryos were lysed in 10 mM Tris pH 7.0 containing 1% Triton X-100. Following BCA-based protein quantitation, equal amounts of protein were run under nonreducing conditions on SDS-PAGE zymography gels containing 0.33% gelatin. In-gel renaturation was performed with 2.5% Triton X-100 followed by 4- or 16-hour incubation in zymogram development buffer (catalog 1610766, Bio-Rad). Gels were stained using Coomassie Brilliant Blue R-250 (Bio-Rad).

### Flow cytometry and N-cadherin surface expression.

Genotyped *fli1a*:EGFP embryos were dissociated as previously described (55). Cell surface N-cadherin was stained as described and analyzed on a Beckman Coulter CytoFLEX Flow Cytometer.

### Pharmacological inhibitors.

Proprotein convertases were globally inhibited using chloromethylketone (catalog ALX-260-022, Enzo). Embryos were treated as indicated in Results by injecting 1.25 μM PCI (solubilized in 50% DMSO) into the pericardial space of live embryos. Control embryos were similarly injected with 50% DMSO. For experiments involving sulforaphane, either 45 μM sulforaphane (LKT Laboratories) or DMSO (final concentration of 0.01%) was added to the embryo medium 3 dpf.

### Fluorophore-assisted carbohydrate electrophoresis.

LLOs, free glycans, and sugar monophosphates were isolated from methanolic sonicates of embryos (9) and analyzed by fluorophore-assisted carbohydrate electrophoresis (FACE). LLO glycans were released from dolichol with mild acid. Glycans were labeled with 7-amino-1,3-naphthalenedisulfonic acid (AnaSpec catalog 81529), monosaccharides were labeled with 2-aminoacridone (MilliporeSigma catalog 06627), and samples were normalized to total cellular protein for FACE analysis. Gel images were acquired with a UVP/Analytik Jena Chemidoc-It II scanner equipped with a model 315 CMOS camera and VisionWorks software.

### MALDI MS imaging.

N-glycan imaging studies were performed as previously described (37, 59, 60) with modifications for freshly frozen, embedded zebrafish. Initial washing included a triple rinse in Carnoy’s solution to remove lipids and metabolites and a rinse in ammonium formate to remove embedding medium (61). Enzyme spraying was adjusted to 50 μL/min to maintain high spatial resolution localization. To minimize matrix clusters, 5 mM ammonium phosphate was sprayed onto the tissues at 2 passes at 50 μL/min, velocity 1300 mm/min, 65°C, 10 psi, and 3 mm offset. Imaging was done on a MALDI-Qtof (timsTOF fleX, Bruker) in positive ion mode over *m/z* 700–300. Laser step size was 20 μm with spot diameter adjusted to 6 μm. A minimum of 5 zebrafish per group were collected in sequence individually and uploaded into SCiLS Lab version 9.00.12376 (Bruker). Data were normalized to total ion current, and N-glycan *m/z* peak intensity over each zebrafish was exported for analysis as peak intensity and analyzed in GraphPad Prism version 9.1.0, reporting mean peak intensity and standard deviation. Results are expressed as mean ± standard deviation. The Mann-Whitney *U* test *P* < 0.01 and < 0.001 were used to report significant differences in regulation between groups.

### Statistics.

All experiments involving analyses of phenotypic rescue were single blinded such that the person acquiring and analyzing the measurements was unaware of the experimental condition. All results are expressed as mean ± SEM, except for MALDI MS results, which are expressed as mean ± standard deviation. Statistical analyses were performed on GraphPad Prism (Version 7.0a) software. For paired comparison of 2 groups, a 2-tailed paired Student’s *t* test was performed. For other parametric data, a 2-way ANOVA was performed, followed by Dunnett’s multiple comparisons test.

### Study approval.

Handling and euthanasia of fish for all experiments were in compliance with policies of the Greenwood Genetic Center, as approved by the GGC Institutional Animal Care and Use Committee (permit A2019 01-003-Y2-A2).

## Author contributions

HFS, RS, HHF, and EJK conceived the study. HFS, RS, EJK, MAL, and HHF designed methodology. EJK, LDR, BK, ZJX, TD, MAL, PA, and RRD investigated. HFS visualized data. HFS, RS, PA, RRD, MAL, and HHF supervised. HFS and RS wrote the original draft. HFS, RS, HHF, MAL, and PA reviewed and edited the draft.

## Supplementary Material

Supplemental data

## Version 1 11/16/2021

In-Press Preview

## Version 2 12/22/2021

Electronic publication
