# Supplementary material for: Protease-dependent defects in N-cadherin processing drive PMM2-CDG pathogenesis
Source: JCI Insight. 2021 Dec 22;6(24):e153474. doi: 10.1172/jci.insight.153474 (PMC8783681; doi:10.1172/jci.insight.153474)
Supplement: Supplemental data [file jciinsight-6-153474-s020.pdf]

Supplementary Materials for

Protease-dependent defects in N-cadherin processing drive PMM2-CDG pathogenesis

Elsenoor J. Klaver[2$], Lynn Dukes-Rimsky[1$], Brijesh Kumar[1], Zhijie Xia[3], Tammie Dang[4], Mark A. Lehrman[4], Peggi Angel[5], Richard R. Drake[5], Hudson H. Freeze[3], Richard Steet[1,2], and Heather Flanagan-Steet[1,2*]

*Corresponding author. Email: heatherfs@ggc.org

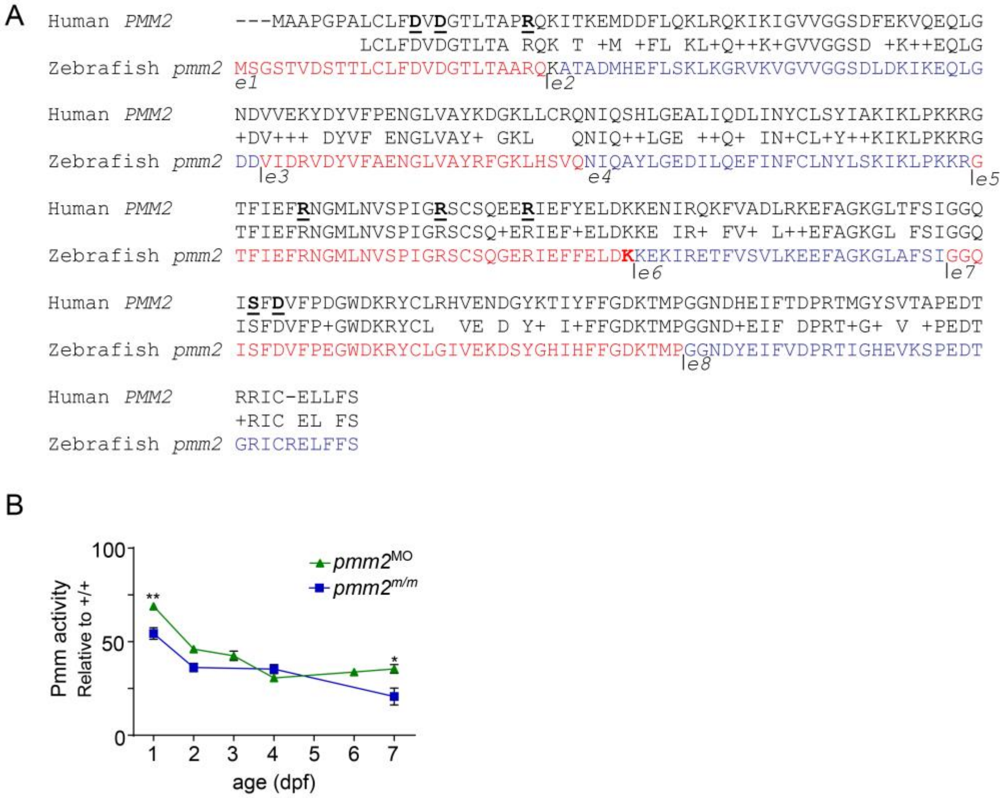

Fig. S1.

A Alignment between zebrafish and human pmm2 enzymes. Bolded underlined residues comprise the substrate binding site, two of which are lost in the one functional *pmm2* transcript made in mutants. Bolded red residue indicates site of the zebrafish mutation

B . Graph compares Pmm2 enzyme activity in morphant and mutant embryos 1-7dpf

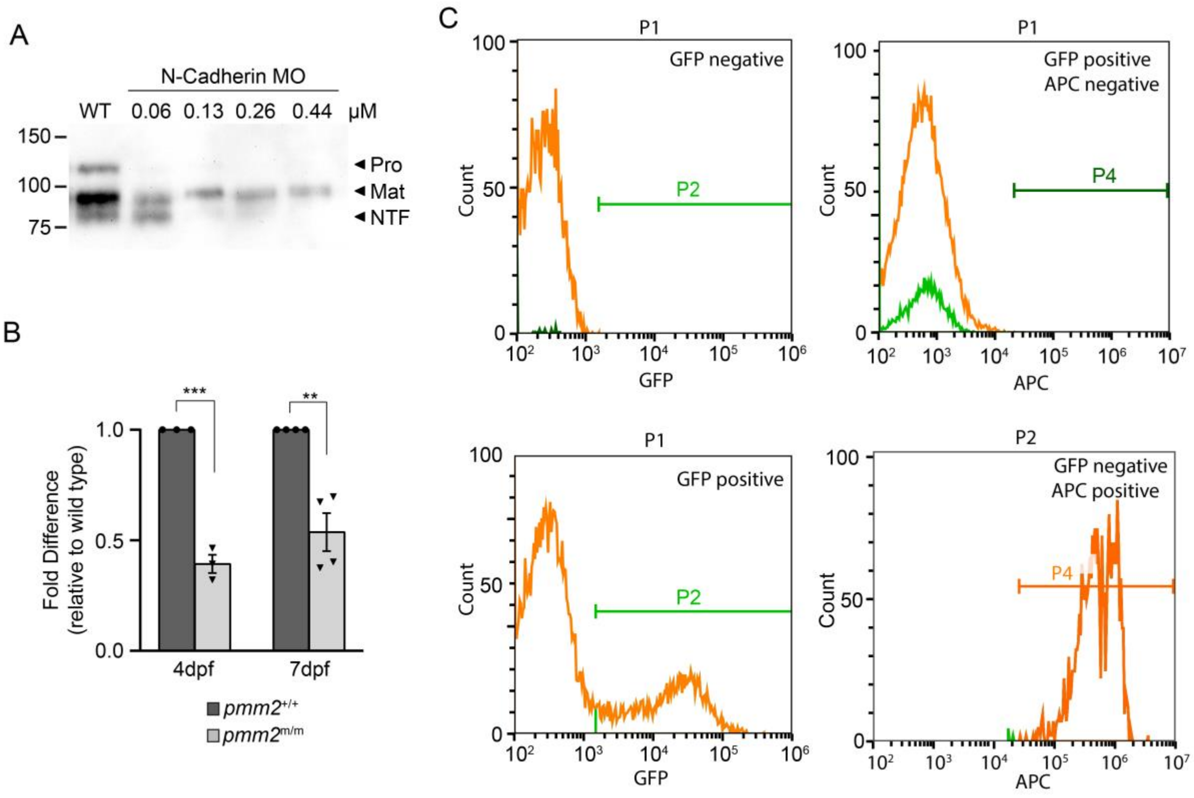

Fig. S2.

A Representative western blot of N-cadherin knockdown in 4dpf embryo lysates show antibody specifically recognizes the pro, mature (Mat), and N-terminal fragments.

B Quantitative RT-PCR of N-cadherin transcript abundance show steady state levels are reduced in *pmm2*$^{m/m}$ embryos compared to *pmm2*$^{+/+}$.

C Representative plots from Flow Cytomtery analyses of cell surface N-cadherin show controls for gate selection of GFP+ and N-cadherin+ cell populations.

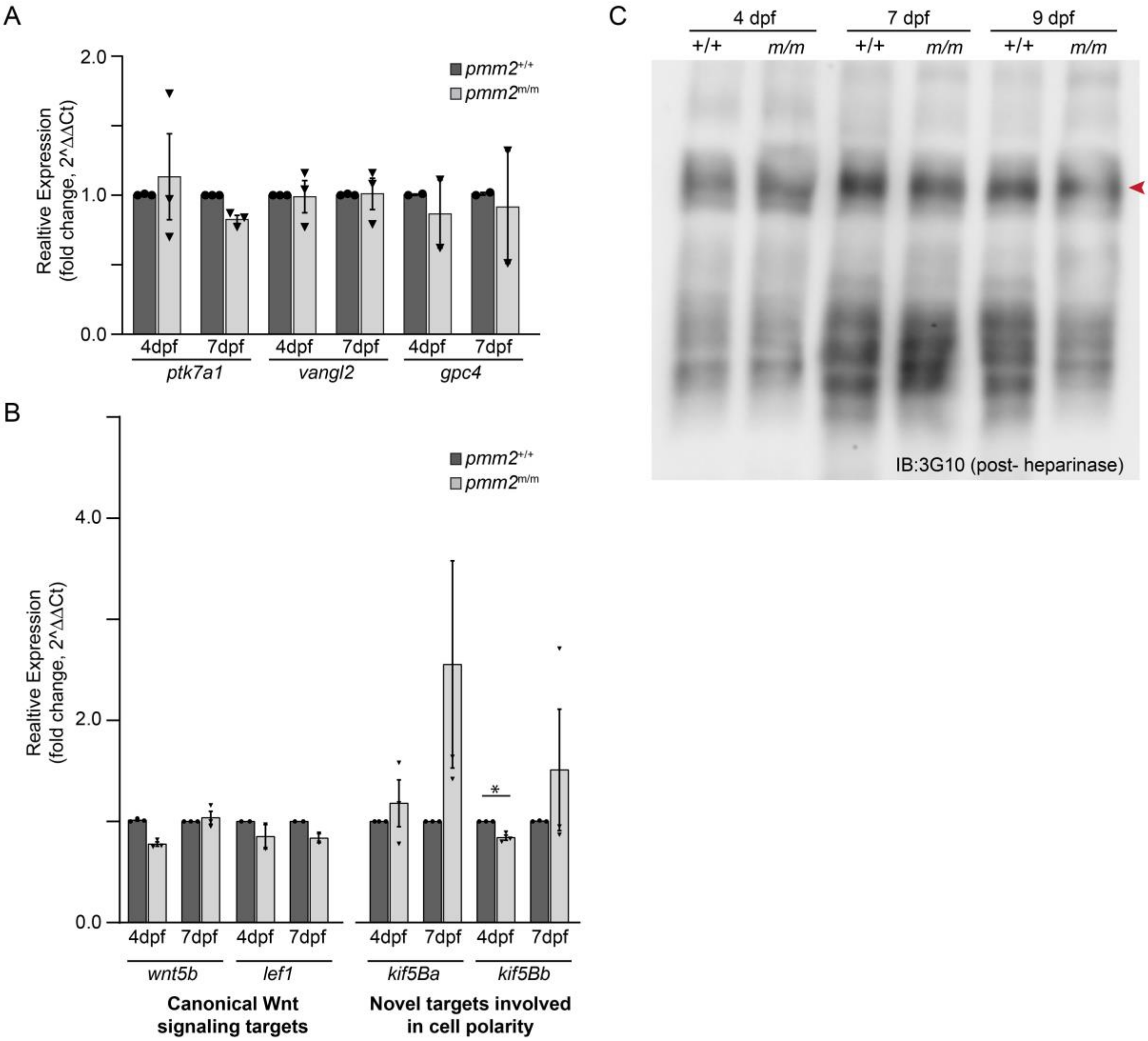

Fig. S3

A,B Quantitative RT-PCR of transcript abundance of several targets of (A) non-canonical or (B) canonical Wnt show steady state levels are not statistically altered by *pmm2* deficiency. The one exception is two novel genes involved non-Wnt mediated cell polarity, *kif5Ba* and *kif5Bb*, which are highly variable in *pmm2*$^{m/m}$ embryos.

C Representative western blot of global glypican analyses in embryos 4,7,9dpf show very little difference in global abundance between *pmm2*$^{+/+}$ and *pmm2*$^{m/m}$ embryos.

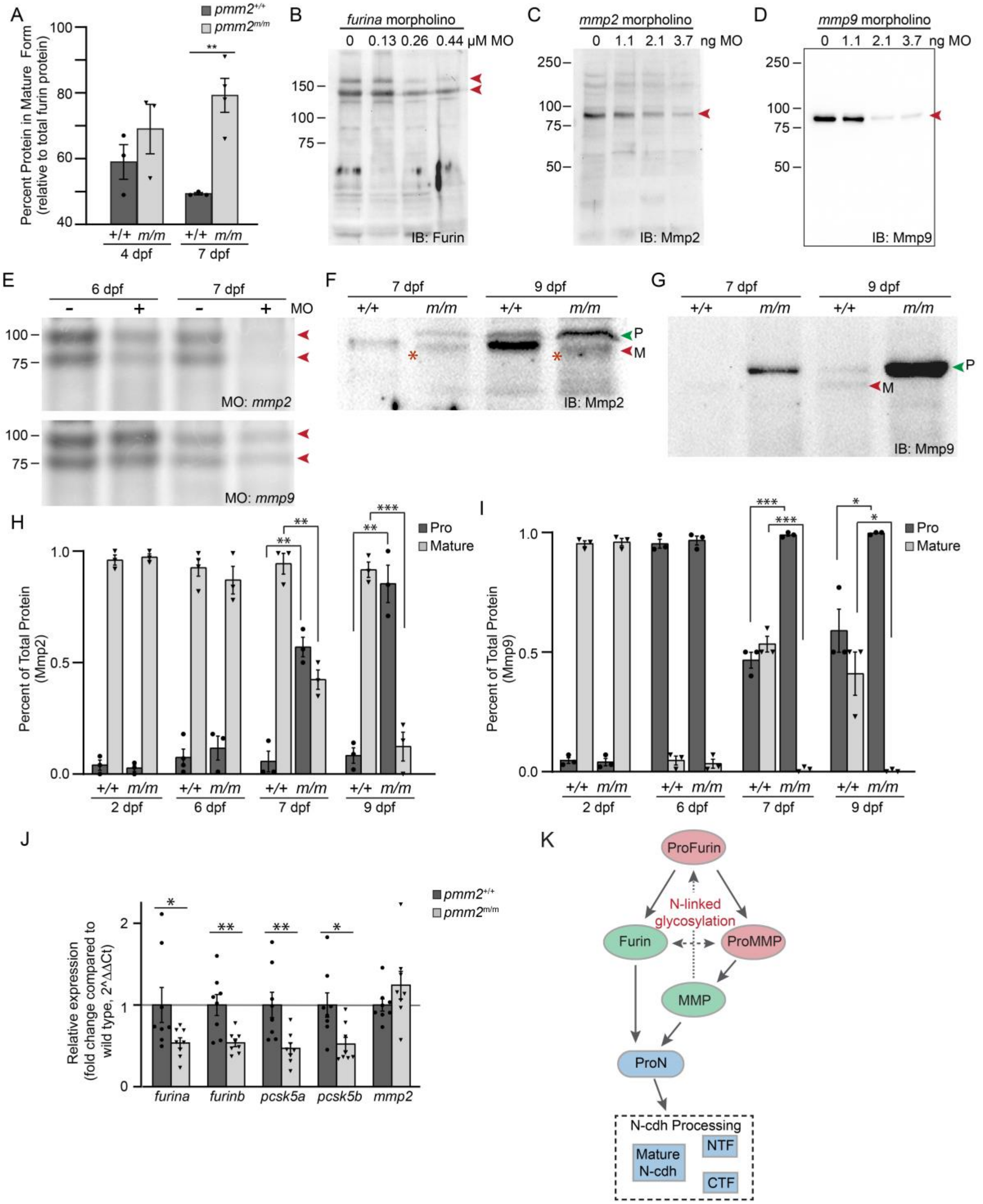

Fig. S4.

A Graph quantitating the percent of total furin protein present in the mature form on western blots from embryonic lysates shows increased abundance of mature furin relative

to total protein at 4 and 7dpf in *pmm2* mutants. n=3 experiments of 15 embryos per sample per experiment. Error=SEM, Student's t-test, **p<0.01

B Representative western blot of *furina* morpholino-mediated knockdown in 4dpf embryo lysates show antibody specifically recognizes protein bands consistent with pro and mature forms.

C Representative western blot of *mmp2* morpholino-mediated knockdown in 4dpf embryo lysates show antibody specifically recognizes protein bands consistent with pro and mature forms.

D Representative western blot of *mmp9* morpholino-mediated knockdown in 4dpf embryo lysates show antibody specifically recognizes protein bands consistent with mature form.

E Gelatin zymography of 6 and 7dpf embryo lysates in which either *mmp2* or *mmp9* expression was morpholino inhibited show Mmp2 contributes the majority of gelatinase activity at these stages. Red arrows highlight key activities.

F,G Enlarged images of western blots shown in Fig 4. Pro (P) and mature (M) forms of either Mmp2 or Mmp9 are indicated with arrows. The shift in size of mature Mmp2 (noted at both 7 and 9dpf) is indicated with red stars

H,I Graph quantitating the percent of total protein present in the Pro (dark box) or Mature (light box) form of either Mmp2 or Mmp9 on western blots of $pmm2^{+/+}$ and $pmm2^{m/m}$ embryonic lysates 2-9dpf. n=3 experiments of 15 embryos per sample per experiment. Error=SEM, Student's t-test, *p<0.05,**p<0.01, ***p<0.001

J Quantitative RT-PCR of transcript abundance of multiple protein proconvertases and mmp2 show steady state levels of several enzymes is reduced in $pmm2^{m/m}$ embryos compared to $pmm2^{+/+}$. n=5-6 experiments of 10-15 embryos per sample per experiment. Error=SEM, Student's t test, *p<0.05,**p<0.01.

K Schematic summarizes model of interactions between PCs (like furin), Mmps, and N-cadherin indicating that N-glycosylation impacts this relationship and N-cadherin processing.

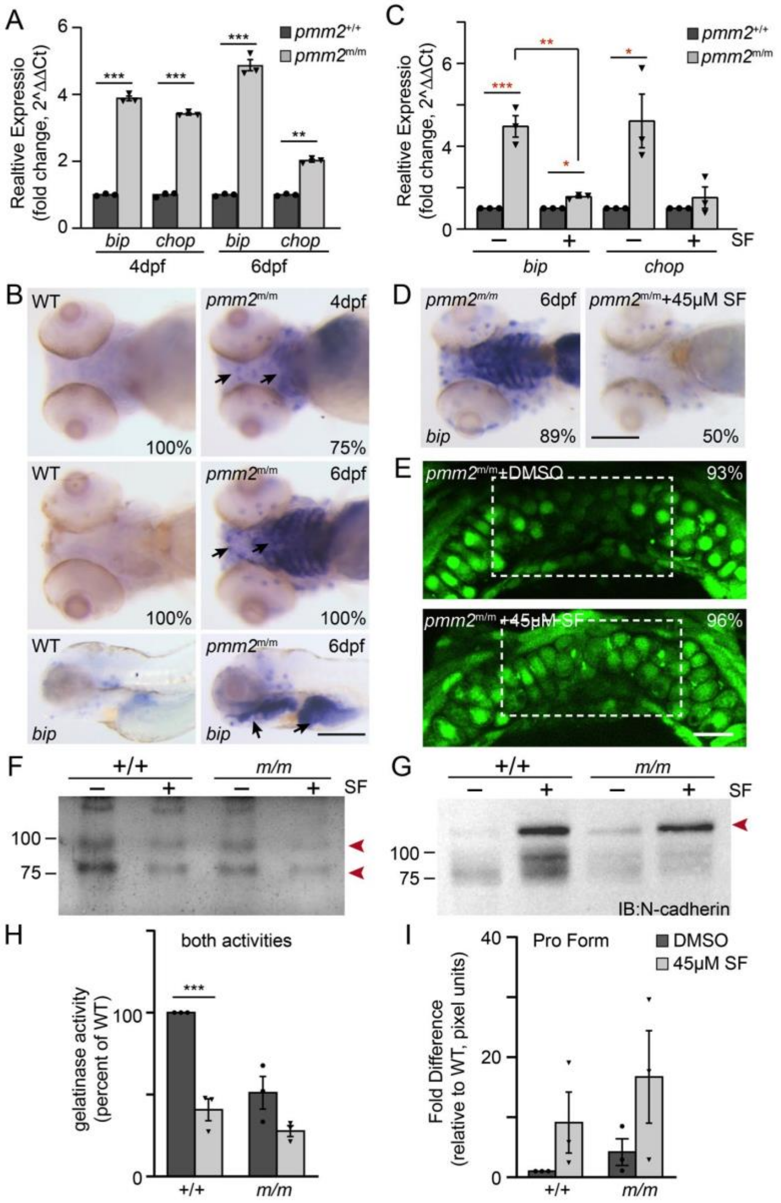

Fig. S5. **Sulphorophane treatment does not improve cartilage phenotypes.** A) qPCR analyses of *bip* and *chop* transcript abindance at 4 and 6 dpf. n=3 experiments with 15 embryos per sample. Error=SEM, Student's t-test, **p<0.01, ***p<0.001. B) In situ hybridization for *bip* 4 and 6dpf. Black arrows highlight increased staining in *pmm2*$^{m/m}$ embryos. Percent values indicate the number of scored embryos exhibiting pictured phenotype. n=15-20 embryos over 4

experiments. Scale bars= 100µm. C) qPCR analyses of *bip* and *chop* transcript abundance at 6 dpf show SF treatment reduces their abundance. n=3 experiments with 15 embryos per sample. Error=SEM, Dunnett's test, *p<0.05, ***p<0.001. D) In situ hybridization for *bip* 6dpf show SF locally decreases transcript abundance. Percent values indicate the number of scored embryos exhibiting pictured phenotype. n=15-20embryos over 3 experiments. Scale bars = 100µm. E) Confocal analyses of *fli1a*:EGFP positive cartilages show no improvement following SF treatment. Percent values indicate the number of scored embryos exhibiting pictured phenotype. n=16 embryos over 3 experiments. Scale bars = 10µm. F) In gel zymography shows SF treatment further reduces gelatinase (Mmp activity in both *pmm2*$^{+/+}$ and *pmm2*$^{m/m}$ embryos. Red arrows denote key activites mediated by Mmp2. n=3 experiments with 15 embryos per sample per experiment. G) Graphic quantitation of zymography analyses of SF treatment. n=3 experiments with 15 embryos per sample per experiment. Error=SEM, Student's t-test, ***p<0.001.H) Representative western blot of N-cadherin processing shows SF treatment increases abundance of Pro N-cadherin in both *pmm2*$^{+/+}$ and *pmm2*$^{m/m}$ embryos. n=3 experiments. I) Graphic quantitation of western blot analyses of Pro N-cadherin in SF treated embryos. . n=3 experiments with 15 embryos per sample per experiment. Error=SEM, Student's t-test, ***p<0.001

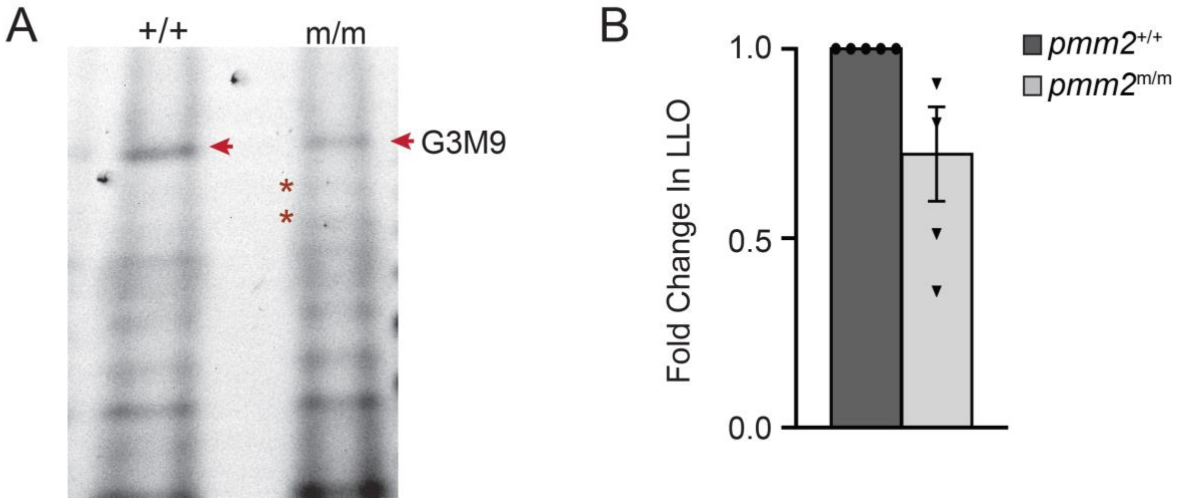

Fig. S6. LLO levels trend down in *pmm2* mutants but the reduction is not statistically significant.

A Representative gel of LLO analyses by FACE. Red asterisks highlight increased abundance of two species, likely assembly intermediates, in *pmm2*$^{m/m}$ embryos

B Quantitation of LLO abundance in *pmm2*$^{+/+}$ and *pmm2*$^{m/m}$ embryos 6dpf.

qPCR	*mmp2* for	GGCGAGTTCTGTAAGTTTCCA
	mmp2 rev	TCATCTCGACCCTGAGAGGT
	mmp9 for	ATCTGTGTTCGTGACGTTTCC
	mmp9 rev	CATCCGCTAGCTGTGTGTTG
	mmp13 for	TCCAGCGATGTGAGTCTGAG
	mmp13 rev	TCCAGCTTTCCAGACACCT
	mmp2 for	CATGGATGGAGAAGCCGACA
	mmp2 rev	AGCAGACCATCCTTGCCATC
	furina for	CACCGATCCCAAGTTTGCAC
	furina rev	GACACGACGACTCCTTGTCC
	furinb for	ATGGTTTCGTCAGTCACGGG
	furinb rev	TGAACCCCTCTATGGCTGGA
	furina for	CGATGTTAACGATGGCGACC
	furina rev	CTATTGGTGTAGCCGTCGCA
	pcsk5a for	AACCGGTGGGCAGTCAAAAT
	pcsk5a rev	CACTAGCCCACCTATCTGGC
	pcsk5a set 2 for	AGCCGTGTCCTTCATACTGC
	pcsk5a set 2 rev	TGGGTCTTCCACCTCTCCTT
	pcsk5b for	TCACTGTTCATGCGACGGTT
	pcsk5b rev	TCAAGATACCAGGGCTTGCG
	mmp2 for	GGCGAGTTCTGTAAGTTTCCA
	mmp2 rev	TCATCTCGACCCTGAGAGGT
	mmp9 for	ATCTGTGTTCGTGACGTTTCC
in situ		
	bip for	GTGTAAAACGACGGCCAGTAG
	bip rev	TTCACTTACCTACTGGGCCC
	chop for	GTGTAAAACGACGGCCAGTAG
	chop rev	TTCACTTACCTACTGGGCCC

Supplemental Table I. Sequences of primers used in this study.
